# Siglec-F^+^ neutrophils in the spleen induce immunosuppression following acute infection

**DOI:** 10.7150/thno.93812

**Published:** 2024-04-08

**Authors:** Chaoxiong Liao, Shuhua Luo, Xiaolei Liu, Lina Zhang, Pengyun Xie, Wending Zhou, Yue Lu, Hanhui Zhong, Xuedi Zhang, Ziying Xiong, Xiao Huang, Guixi Mo, Daqing Ma, Jing Tang

**Affiliations:** 1 Department of Anesthesiology, Affiliated hospital of Guangdong Medical University, Zhanjiang 524000, Guangdong, China.; 2 Guangdong Medical University, Zhanjiang 524000, Guangdong, China.; 3 Division of Anaesthetics, Pain Medicine and Intensive Care, Department of Surgery and Cancer, Faculty of Medicine, Imperial College London, Chelsea and Westminster Hospital, London, UK.; 4 Children's hospital, Zhejiang University School of Medicine, National Clinical Research Center for Child Health, Hangzhou, China.

**Keywords:** Sepsis, Immunosuppression, Secondary challenge, Siglec-F, Neutrophils, T lymphocytes, IL-10

## Abstract

**Background:** The mechanisms underlying the increased mortality of secondary infections during the immunosuppressive phase of sepsis remain elusive.

**Objectives:** We sought to investigate the role of Siglec-F^+^ neutrophils on splenic T lymphocytes in the immunosuppressed phase of sepsis and on secondary infection in PICS mice, and to elucidate the underlying mechanisms.

**Methods:** We established a mouse model of sepsis-induced immunosuppression followed by secondary infection with LPS or *E. coli.* The main manifestation of immunosuppression is the functional exhaustion of splenic T lymphocytes. Treg depletion reagent Anti-IL-2, IL-10 blocker Anti-IL-10R, macrophage depletion reagent Liposomes, neutrophil depletion reagent Anti-Ly6G, neutrophil migration inhibitor SB225002, Siglec-F depletion reagent Anti-Siglec-F are all used on PICS mice. The function of neutrophil subsets was investigated by adoptive transplantation and the experiments in vitro.

**Results:** Compared to other organs, we observed a significant reduction in pro-inflammatory cytokines in the spleen, accompanied by a marked increase in IL-10 production, primarily by infiltrating neutrophils. These infiltrating neutrophils in the spleen during the immunosuppressive phase of sepsis undergo phenotypic change in the local microenvironment, exhibiting high expression of neutrophil biomarkers such as Siglec-F, Ly6G, and Siglec-E. Depletion of neutrophils or specifically targeting Siglec-F leads to enhance the function of T lymphocytes and a notable improvement in the survival of mice with secondary infections.

**Conclusions:** We identified Siglec-F^+^ neutrophils as the primary producers of IL-10, which significantly contributed to T lymphocyte suppression represents a novel finding with potential therapeutic implications.

## Introduction

Sepsis, a systemic inflammatory condition triggered by severe infection 1, stands as a significant health challenge. It frequently culminates in multiple organ dysfunction syndrome (MODS) 2, representing a leading cause of mortality in intensive care units (ICUs) 3. Regrettably, many survivors of sepsis experience heightened mortality rates in the months or even years following the initial infection, primarily due to entering a prolonged state of immune suppression 4-7. This phenomenon, known as persistent inflammation, immunosuppression, and catabolism syndrome (PICS), arises from unresolved primary infections or the acquisition of opportunistic pathogens. Managing sepsis effectively encompasses addressing the sequelae of PICS, which pose substantial challenges 8-13.

The spleen, as the largest secondary lymphoid organ, plays a pivotal role in regulating both innate and adaptive immune responses 14. Its immune cell composition is critical for sustaining long-term antigen tolerance. Notably, T lymphocytes within the spleen contribute to immune tolerant development 15, while splenic tumor-associated neutrophils exert influence over tumor growth and metastasis 16. Neutrophils, as specialized immune cell, exhibit remarkable plasticity 17-19 and can differentiate into distinct subsets under various conditions, spanning acute infections, chronic inflammation, and tumor progression 20-23. In light of these considerations, our study aims to elucidate whether specific subsets of infiltrating neutrophils within the spleen contribute to the immunosuppressive phase of sepsis in mice. Furthermore, we seek to unravel the underlying mechanisms driving these phenomena.

## Results

### Immunosuppression state of the spleen with depleted T lymphocytes in PICS and PICS+LPS 6 h mice

**Figure 1:**
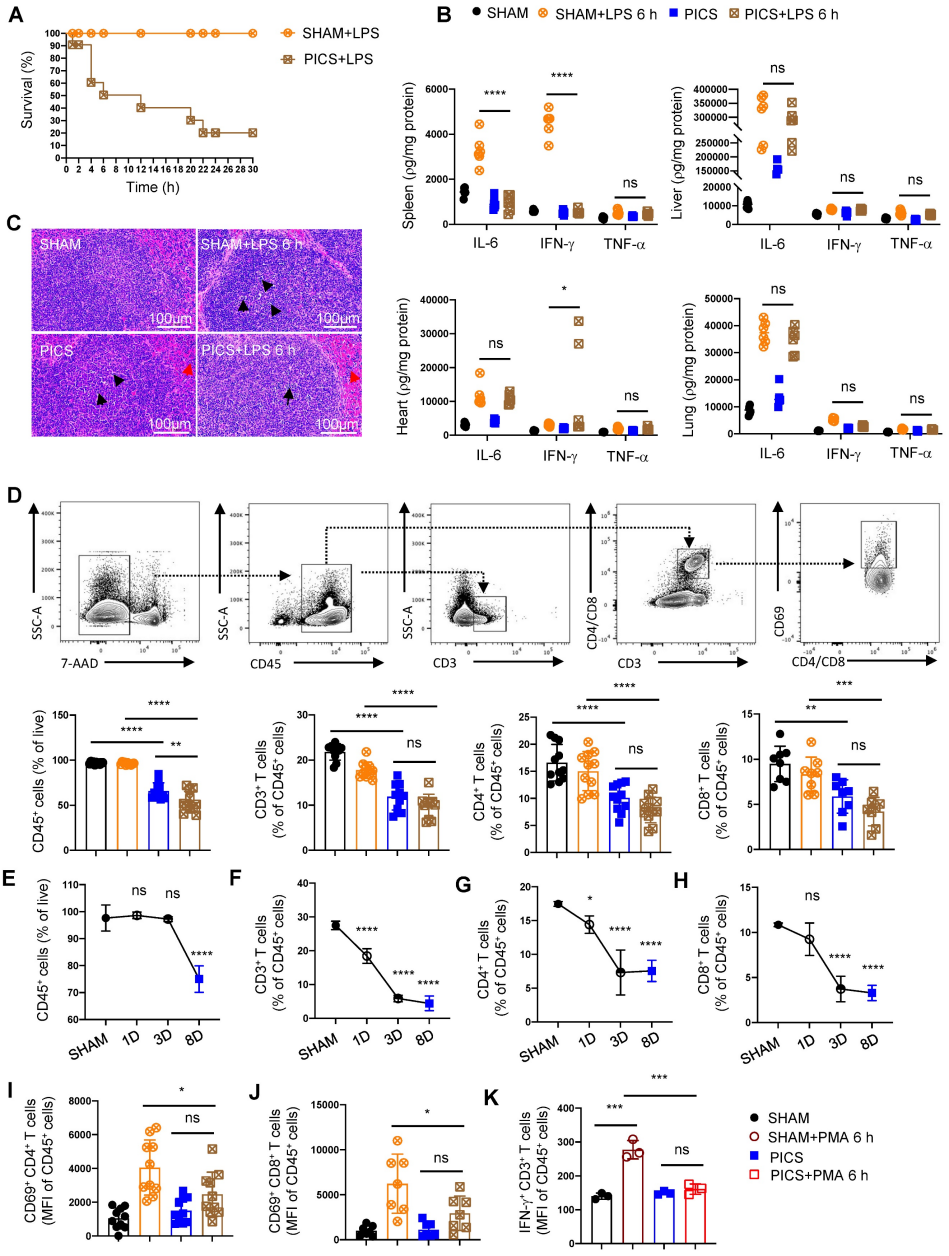
** The immunosuppressive state of the spleen with the depleted proportion and function of T lymphocytes in PICS and PICS+LPS 6 h mice. (A)** SHAM group (n = 10) and PICS group (n = 10) were intraperitoneally injected with LPS (10 mg/kg), and the number of surviving mice after challenge was counted. Survival curves on secondary infections in PICS mice were analyzed compared to the initial insult (SHAM+LPS 6 h). **(B)** Expression of proinflammatory factors (IL-6, IFN-γ, TNF-α) in the spleen, liver, heart, and lung tissues was examined by enzyme-linked immunosorbent assay after 6 h of intraperitoneal injection with LPS or saline in SHAM and PICS mice.** (C)** Spleens from all groups were stained with HE to observe the degree of injury and the changes of immune cells. **(D)** Proportions of CD45^+^ immune cells, CD3^+^, CD4^+^, and CD8^+^ T lymphocytes in the spleens of all groups were measured by flow cytometry. **(E-H)** Proportion of CD45^+^ immune cells, CD3^+^, CD4^+^, and CD8^+^ T lymphocytes in the spleens on SHAM and the first, third, eighth day after CLP were detected by flow cytometry. **(I-J)** Activity of CD4^+^ and CD8^+^ T lymphocytes in the spleens of all groups were measured by flow cytometry. **(K)** Before examining the expression of IFN-γ, the CD3^+^ T lymphocytes in the spleens were sorted by flow cytometry and stimulated in vitro for 6 h with phorbol 12-myristate-13-acetate (PMA, 50 ng/ml) and ionomycin (1 μg/ml) in the presence of brefeldin A (0.5 μl/ml). One-way ANOVA followed by Dunnett's post hoc test was applied in **(B, D-J)**. Data are presented as mean ± SEM. n = 3-14, *ns P* >0.05, *P <0.05, **P < 0.01, ***P < 0.001, ****P < 0.0001.

Secondary infections represent a major contributor to mortality in late-stage septic patients, yet the underlying molecular mechanisms remain poorly understood. To address this gap, we established a mouse model of “PICS” and simulated “secondary infection” by intraperitoneal injection of endotoxin (LPS, 10 mg/kg). In the spleen, bone marrow and peripheral blood of PICS mice, we observed an increased proportion of macrophages and neutrophils (Figure S1A-D). Conversely, the proportion of CD4^+^ and CD8^+^ T lymphocytes was decreased, accompanied by upregulation of the immune inhibitory receptor PD-L1 (Figure S1E-H), indicative of an immunosuppressed state. Furthermore, the functionality of IFN-γ-expressing T lymphocytes was impaired (Figure 1K), while Treg activity and IL-10 expression in CD45^+^ immune cells were elevated (Figure S1I-J). Additionally, PICS mice displayed significant weight loss and signs of emaciation (Figure S1K), suggestive of heightened catabolic metabolism.

Compared to the initial infection (acute phase), the survival rate of PICS mice significantly declined, reaching only 50% after 6 h of secondary infections and further plummeting to 20% after 24 h (Figure 1A). To elucidate the underlying mechanisms, we examined IL-6, IFN-γ, and TNF-α in spleen, liver, heart, and lung tissues, revealing a significant in their expression levels specifically within the spleens (Figure 1B), consistent with clinical observations in late-stage sepsis patients 13, 24. These finding suggest that the spleen in PICS mice adopts an immunosuppressive phenotype, potentially contributing to the heightened mortality observed during the secondary infections.

Histological analysis via hematoxylin and eosin (HE) staining revealed a decrease in the number of white pulp T lymphocytes in the spleen of PICS mice at 6 h post-LPS challenge (LPS 6 h) compared to SHAM and SHAM+LPS 6 h mice (indicated by black arrows; Figure 1C). Additionally, a notable infiltration of neutrophils was observed (indicated by the red arrows; Figure 1C). These findings were corroborated by flow cytometry data (Figure 1D, 3A). Furthermore, we observed a significant reduction in the proportion of CD45^+^ cells in PICS mice, which was further decreased following secondary infections (Figure 1D). Moreover, the proportion of CD45^+^ cells began to decline gradually from the third day onwards, indicative of early immune suppression (Figure 1E). Consistent with previous reports suggesting inflammatory stimuli induce lymphocyte apoptosis 25, our study demonstrated a decrease in the proportions of CD3^+^, CD4^+^, and CD8^+^ T lymphocytes as early as the first day following CLP surgery (Figure 1F-H). Notably, the activity of CD4^+^ and CD8^+^ T lymphocytes was significantly suppressed in the PICS+LPS 6 h group compared to the SHAM+LPS 6 h group (Figure 1I-J).

Further examination of changes in other immune cell populations revealed a decrease in the proportion of the lymphocytes and monocytes, coupled with a significant increase in granulocytes in both the PICS and PICS+LPS 6 h groups (Figure S2A). The increase in granulocytes was primarily driven by CD11b^+^ CD45^+^ cells in the spleen, the peripheral blood, and bone marrow (Figure S2B), likely reflecting persistent inflammatory stimulation leading to the recruitment of myeloid immune cells to the peripheral immune organs. While B cells, NK cells, and CD31^+^ macrophages have been reported to possess anti-inflammatory and immunosuppressive effects 26-28, we observed no significant differences in these cell populations (Figure S2C-D, J). Notably, despite previous reports highlighting the interplay between Th1 cells and Tregs, and Th17 cells and Treg cells 29,30, 31, we found no significant difference in the proportions of Th1, Th2, and Th17 cells, nor in the proportion of CD62L^+^ CD4^+^ and CD62L^+^ CD8^+^ T lymphocytes (Figure S2E-I). Overall, our findings suggest that the heightened mortality rate observed during secondary infections may be closely associated with impaired function and reduced number of T lymphocytes in the spleen.

### IL-10 suppresses the immune response in the spleen

Our findings revealed an increase in the fluorescence intensity of FoxP3 in the spleens of PICS mice, indicative of enhanced Treg activity (Figure S1I). Additionally, we observed elevated expression of IL-10 in the spleen (Figure S1J). Furthermore, following LPS treatment, there was a significant increase in the proportion of Treg and the expression of ICOS on Treg cells (Figure S3A-B). Given that Treg are known to suppress the function of T lymphocytes through IL-10 32, 33, we hypothesized their involvement in regulating the splenic immune response. To investigate this, we depleted Treg in PICS mice and subsequently subjected them to LPS challenge (Figure S3C). Interestingly, we observed a decrease in CD45^+^ cells and IL-10 expression in the spleen (Figure S3D-E), while no differences were found in the levels of IL-6, IFN-γ, and TNF-α (Figure S3F). This suggested that Treg did not play a significant role in regulating the splenic immune response in PICS mice. To further exclude the influence of other lymphocyte subsets, such as Tr1 type Treg, we assessed the proportion of lymphocytes secreting IL-10 and found a significant decrease in CD4^+^ and CD8^+^ T lymphocytes after secondary challenge (Figure S3G), resulting in insufficient secretion of IL-10 to inhibit the inflammatory response. Moreover, we observed no significant difference in the expression of TGFβ in CD45^+^ immune cells (Figure S3H). In summary, we indirectly ruled out the effect of lymphocytes subsets on the inflammatory response of the secondary infections.

**Figure 2:**
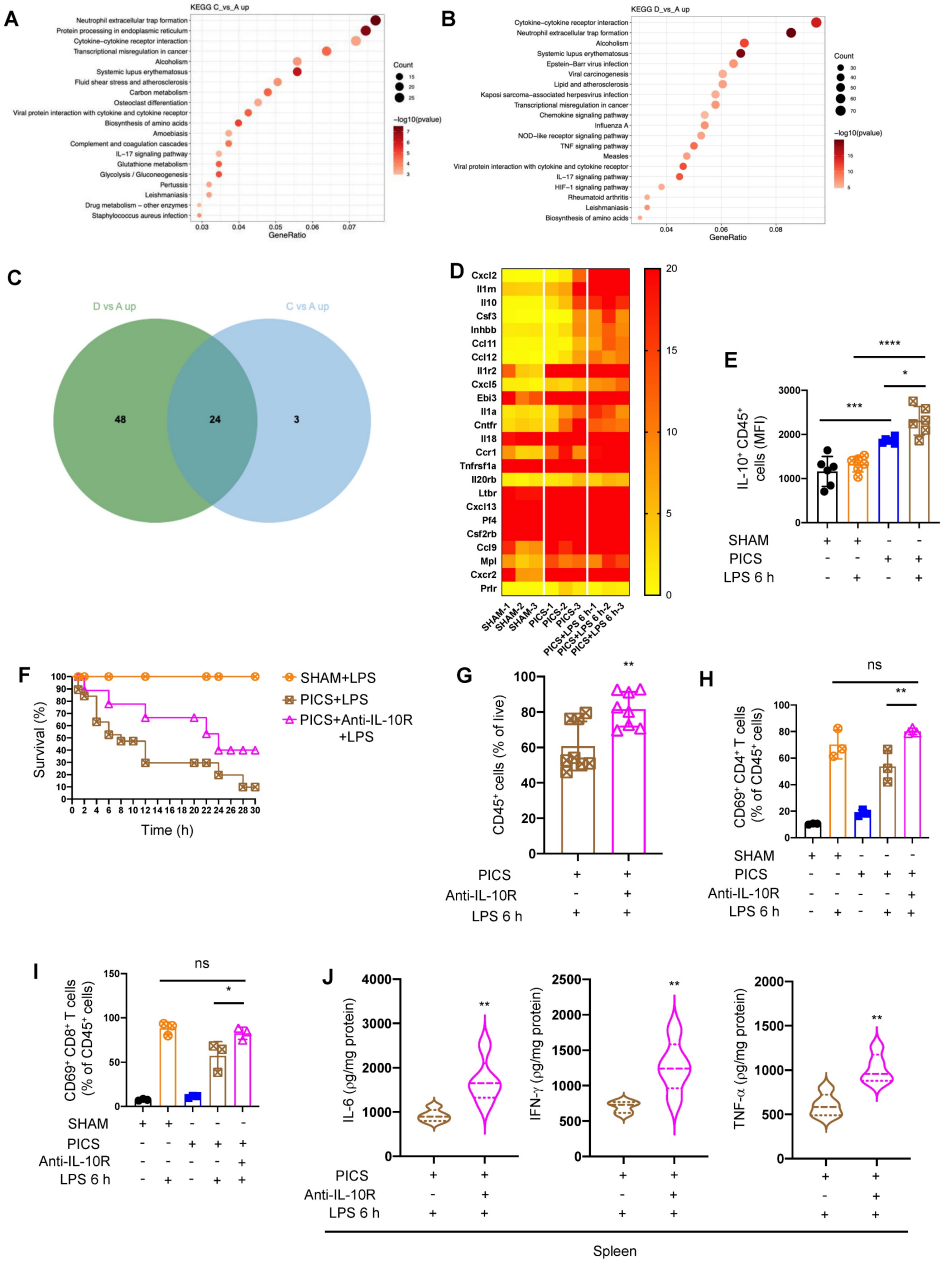
** The results of spleen RNA-seq indicated that the secretion of IL-10 in immune cells inhibited the immune response of spleens. (A-B)** Results of spleens regarding differential gene enrichment pathways of PICS (C) vs SHAM (A) and PICS+LPS 6 h (D) vs SHAM in RNA-seq. **(C-D)** Heat map showing the relative expression of co-upregulated differential genes screened by Wayne analysis in the PICS and PICS+LPS 6 h groups. **(E)** Flow cytometry was used to detect the expression of IL-10 in CD45^+^ immune cells. **(F)** PICS mice were intraperitoneally injected with LPS after treatment with Anti-IL-10R (n = 11) and compared for survival with PICS+LPS (n = 19) mice and SHAM+LPS (n = 7). **(G-J)** After blocking the function of IL-10, the proportions of CD45^+^ immune cells and activity of CD4^+^, CD8^+^ T lymphocytes in the spleens were examined with intraperitoneally injecting LPS by flow cytometry as well as changes in expression of pro-inflammatory cytokines (IL-6, IFN-γ, TNF-α) were detected by enzyme-linked immunosorbent assay. One-way ANOVA followed by Dunnett's post hoc test was applied in **(E, H-I)**. Student's *t* test was applied in** (G, J)**. Data are presented as mean ± SEM. n = 3-19, *ns P* >0.05, *P <0.05, **P < 0.01, ***P < 0.001, ****P < 0.0001.

To elucidate the molecular mechanisms underlying the immune hypo-responsiveness, we conducted RNA-seq analysis on spleen samples harvested from SHAM, PICS, and PICS+LPS 6 h mice. Differentially expressed genes in comparisons of C vs A (PICS vs SHAM) and D vs A (PICS+LPS 6 h vs SHAM) were highly enriched in the cytokine-cytokine receptor interaction pathway (Figure 2A-B). Venn analysis identified 24 differentially co-expressed genes in PICS and PICS+LPS 6 h mice (Figure 2C). Heatmap analysis of co-expressed genes revealed increased expression of IL-10 in the PICS+LPS 6 h group (Figure 2D), with flow cytometry further confirming higher expression of IL-10 in CD45^+^ cells (Figure 2E). These findings suggested that IL-10 may be involved in regulating the immune response during secondary LPS challenge.

To validate the role of IL-10, we administered Anti-IL-10R in PICS mice followed by LPS stimulation. Blocking IL-10 significantly improved the survival (Figure 2F), increased the proportion of CD45^+^ cells (Figure 2G), enhanced the activation ratio of CD4^+^ and CD8^+^ T lymphocytes in the spleen (Figure 2H-I), and upregulated the expression of IL-6, IFN-γ, and TNF-α (Figure 2J). These data indicated that certain immune cells in the spleen inhibit the immune response through IL-10, leading to suppression of T lymphocyte activity and reduced survival rates following secondary LPS treatment.

### Neutrophil-derived IL-10 inhibits the function and activity of T lymphocytes

**Figure 3:**
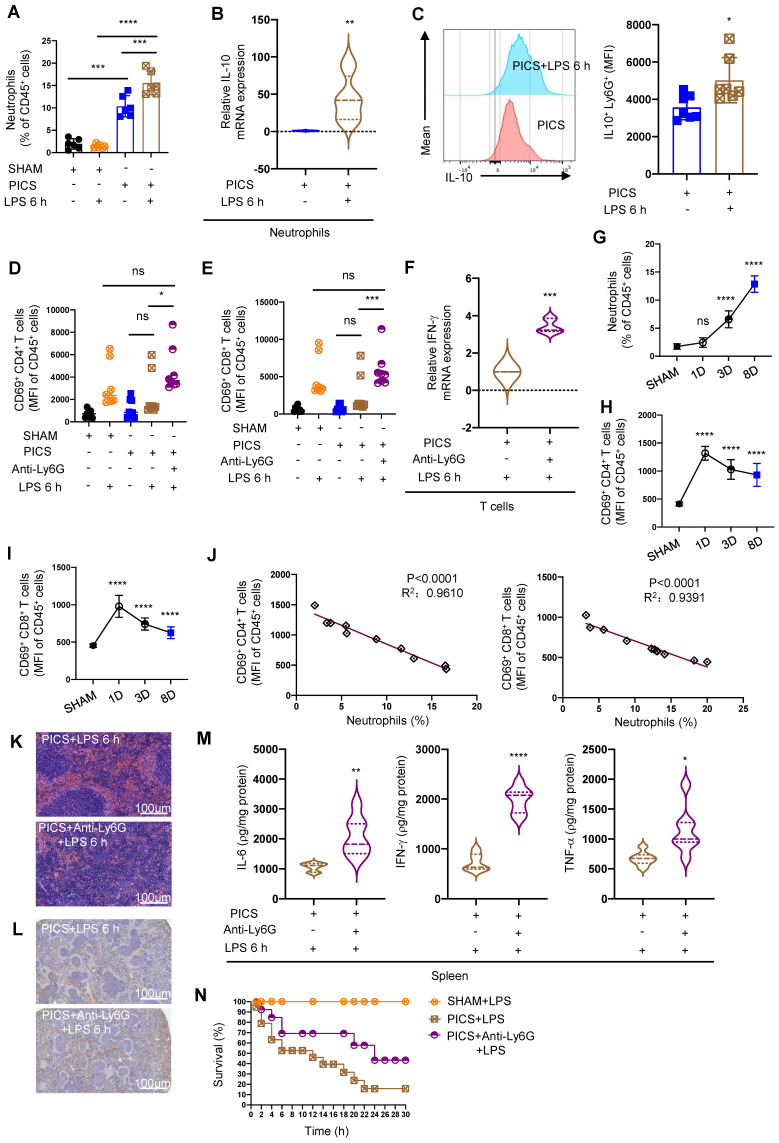
** Splenic neutrophils of PICS mice secreted IL-10 to suppress the immune response of T lymphocytes. (A)** Proportion of neutrophils in the spleens examined by flow cytometry. **(B-C)** Expression of IL-10 in the splenic neutrophils of PICS and PICS+LPS 6 h mice detected by qPCR and flow cytometry. **(D-F)** Activity of CD4^+^ and CD8^+^ T lymphocytes in the spleens detected by flow cytometry, and the relative mRNA expression of IFN-γ in T lymphocytes after flow cytometry sorting examined by qPCR. **(G-I)** Proportion of the splenic neutrophils and the fluorescence intensity of CD69^+^ CD4^+^ and CD69^+^ CD8^+^ T lymphocytes on SHAM and the first, third, eighth day after CLP detected by flow cytometry. **(J)** Correlation between changes in proportions of neutrophils and activity of CD4^+^ and CD8^+^ T lymphocytes demonstrated by correlation analysis. **(K-L)** After depletion of neutrophils, proliferation of splenic T lymphocytes in PICS+LPS 6 h mice observed by HE staining and immunohistochemistry. **(M)** Expression of IL-6, IFN-γ, TNF-α in the spleens detected after depletion of neutrophils in PICS mice injected intraperitoneally with LPS. **(N)** PICS mice intraperitoneally injected with LPS after treated with Anti-Ly6G (n = 14) compared with the survival of the PICS+LPS mice (n = 19) and the SHAM+LPS mice (n = 10). One-way ANOVA followed by Dunnett's post hoc test was applied in **(A, D-E, G-I)**. Student's *t* test was applied in** (B-C, F, M)**. Pearson's correlation test was applied in **(J)**. Data are presented as mean ± SEM. n = 3-19, *ns P* >0.05, *P <0.05, **P < 0.01, ***P < 0.001, ****P < 0.0001.

Our results demonstrated a significant increase in neutrophils in the spleens of PICS and PICS+LPS 6 h mice, as evidenced by HE staining and flow cytometry analysis (Figure 1C, 3A). Moreover, these neutrophils released a substantial amount of IL-10 (Figure S4A). Following LPS stimulation, the levels of IL-10 in neutrophils from PICS mice were markedly elevated (Figure 3B-C), while expression levels of IL-6, IFN-γ, and TNF-α showed minimal increases, indicating neutrophils were in an N2 state (Figure S4B) 34.

To further elucidate the functionality of infiltrated neutrophils in the spleen, we depleted neutrophils (Figure S4C) and observed a reduction in splenic congestion (Figure S4D) and restoration the activity and function of CD4^+^, CD8^+^ T lymphocytes were restored (Figure 3D-F). Moreover, upon secondary infections, IL-10 levels significantly decreased (Figure S5A), accompanied by increased proportions of CD45^+^ cells, CD3^+^, CD4^+^, and CD8^+^ T lymphocytes (Figure S5B-E). Additionally, we observed a gradual increase in neutrophils with statistical differences seen on the third day after CLP surgery (Figure 3G); Additionally, persistent inflammatory stimulation was reported to induce the immune suppression 35. Subsequently, we noted a gradual decrease in CD4^+^ and CD8^+^ T lymphocyte activity after CLP surgery (Figure 3H-I). Importantly we demonstrated a significantly negative correlation between infiltrated neutrophils and of CD4^+^ and CD8^+^ T lymphocytes activity (Figure 3J).

Furthermore, neutrophils depletion enhanced T lymphocyte proliferation (Figure 3K-L), upregulated IL-6, IFN-γ, TNF-α expression (Figure 3M), and significantly improved survival in PICS mice after secondary challenge (Figure 3N). These data suggest that increased neutrophils infiltration in the spleen may be a major contributor to the high mortality of PICS mice upon secondary challenge, and neutrophils depletion reversed T lymphocyte exhaustion.

To further elucidate the role of neutrophils in T lymphocytes through IL-10 secretion, we performed flow cytometry to sort neutrophils and T lymphocytes from the spleens of PICS mice and stimulated them with LPS in vitro. Anti-IL-10R incubation effectively promoted T lymphocytes activation and IFN-γ mRNA expression compared to the Isotype group (Figure S5F-H).

We then investigated the effects of IL-10 secretion from other immune cells on T lymphocytes. While CD3^+^ T lymphocytes were reported to inhibit their own function through autocrine IL-10 secretion 36, CD4^+^ T lymphocytes and NK lymphocytes produced IL-10 during sepsis 27, 30. We found no significant differences in IL-10 expression in CD3^+^, CD4^+^ T lymphocytes, and NK lymphocytes between PICS and SHAM groups (Figure S4E-G). Macrophages were also reported to produce IL-10 to suppress the function of T lymphocytes 37. Our data indicated that, the proportion of macrophages was increased in the spleen of the PICS+LPS 6 h mice (Figure S6A) and there is a significant increase in IL-10 mRNA expression, but it has no statistically significant difference of the IL-10 protein levels (Figure S6B-C). To clarify the impact of macrophages on T lymphocytes, we depleted macrophages in the PICS mice through intraperitoneal injection of liposomes (Figure S6D) and then subjected to LPS challenge. The results showed no significant changes in the proportion of CD45^+^ cells and the activity of CD4^+^, CD8^+^ T lymphocytes (Figure S6E-G). These suggest that macrophages did not contribute in the suppression of T lymphocyte activity. In summary, during the immunosuppressive phase of septic mice, splenic neutrophils suppressed the function and activity of T lymphocytes through significant IL-10 production.

### Inhibition of neutrophil recruitment in the spleen of PICS mice improves the activity of T lymphocytes

**Figure 4:**
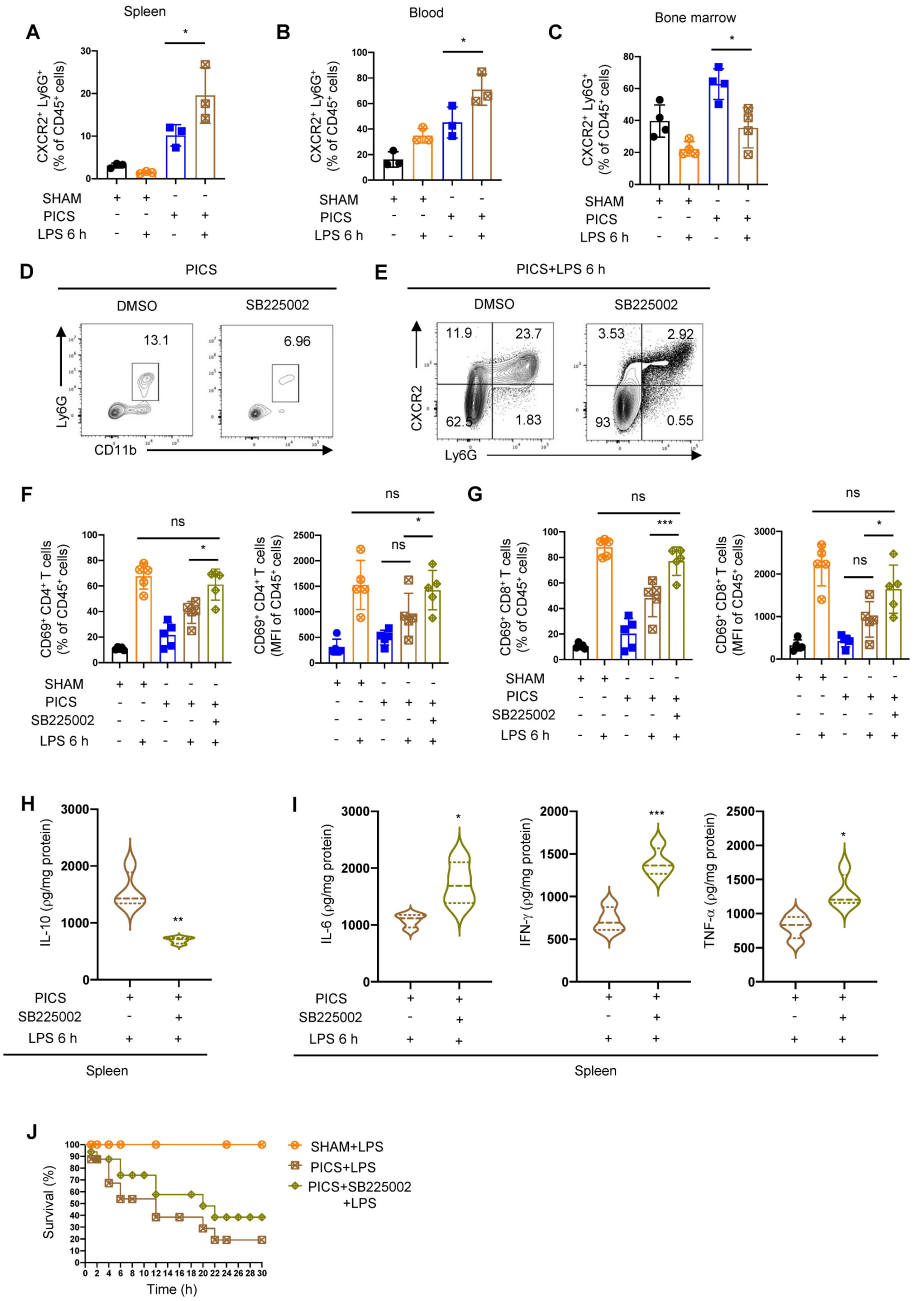
** CXCR2 inhibitor SB225002 can improve the immune response of the spleen. (A-C)** Proportion of CXCR2^+^ neutrophils in the spleen, peripheral blood and bone marrow examined by flow cytometry. **(D)** Efficiency of SB225002 in inhibiting neutrophil recruitment in the spleens. **(E-I)** After treatment of SB225002, proportion of CXCR2^+^ neutrophils and activity of CD4^+^ and CD8^+^ T lymphocytes in the spleens detected by flow cytometry. Expression of IL-10, IL-6, IFN-γ, TNF-α in the spleens of PICS+LPS 6 h and PICS+SB225002+LPS 6 h mice examined by enzyme-linked immunosorbent assay. **(J)** PICS mice intraperitoneally injected with LPS after treated with SB225002 (n = 16), then compared for survival with PICS+LPS mice (n = 16) and SHAM+LPS mice (n = 7). One-way ANOVA followed by Dunnett's post hoc test was applied in **(A-C, F-G)**. Student's *t* test was applied in** (H-I)**. Data are presented as mean ± SEM. n = 3-16, *ns P* >0.05, *P <0.05, **P < 0.01, ***P < 0.001.

To further elucidate the origin of recruited neutrophils in the spleen, we analyzed their sources and observed a significant increase in CXCR2^+^ neutrophils in the spleen and peripheral blood of PICS mice after LPS treatment (Figure 4A-B). In contrast, the proportion of CXCR2^+^ neutrophils in the bone marrow were significantly decreased (Figure 4C). These findings suggested that neutrophils are released from the bone marrow into the peripheral blood, leading to an accumulation of neutrophils in the spleen.

To analyze the functionality of neutrophil recruitment, we pre-treated the mice with the CXCR2 inhibitor SB225002 (Figure 4D) 38 followed by LPS challenge. We identified a significant reduction in CXCR2^+^ neutrophils and increased activation of CD4^+^, CD8^+^ T lymphocytes (Figure 4E-G). Additionally, the expression of IL-10 decreased in the spleen after inhibiting neutrophil recruitment (Figure 4H), accompanied by a significant increase in the expression levels of IL-6, IFN-γ, and TNF-α (Figure 4I). Furthermore, the survival of PICS mice was improved (Figure 4J).

The above data indicated that neutrophils exhibited a high expression of CXCR2. qPCR and ELISA data further showed that T lymphocytes in the spleen of PICS and PICS+LPS 6 h mice exhibited a high expression of CXCL1 and CXCL2 (Figure S7A-B), while the expression levels of other chemokines, such as CXCL3, CXCL5, and CXCL8, did not significant differ compared to the SHAM group (Figure S7C-E). In conclusion, T lymphocytes recruited neutrophils through the secretion of CXCL1 and CXCL2, and the increased recruitment of neutrophils produces a significant amount of IL-10, which then inhibits the function and activity of T lymphocytes.

Additionally, we found that the spleen of PICS+LPS 6 h mice exhibited a high expression of CXCL2, while the expression levels of CXCL1, CXCL3, and CXCL5 were not statistically significant (Figure S7F-I). This suggested that other immune cells, such as macrophages exhibiting high expression of CXCL2 (Figure S7J), may secrete chemokines contributing to neutrophil recruitment.

### LPS induces high expression of Siglec-F in splenic neutrophil of PICS mice

**Figure 5:**
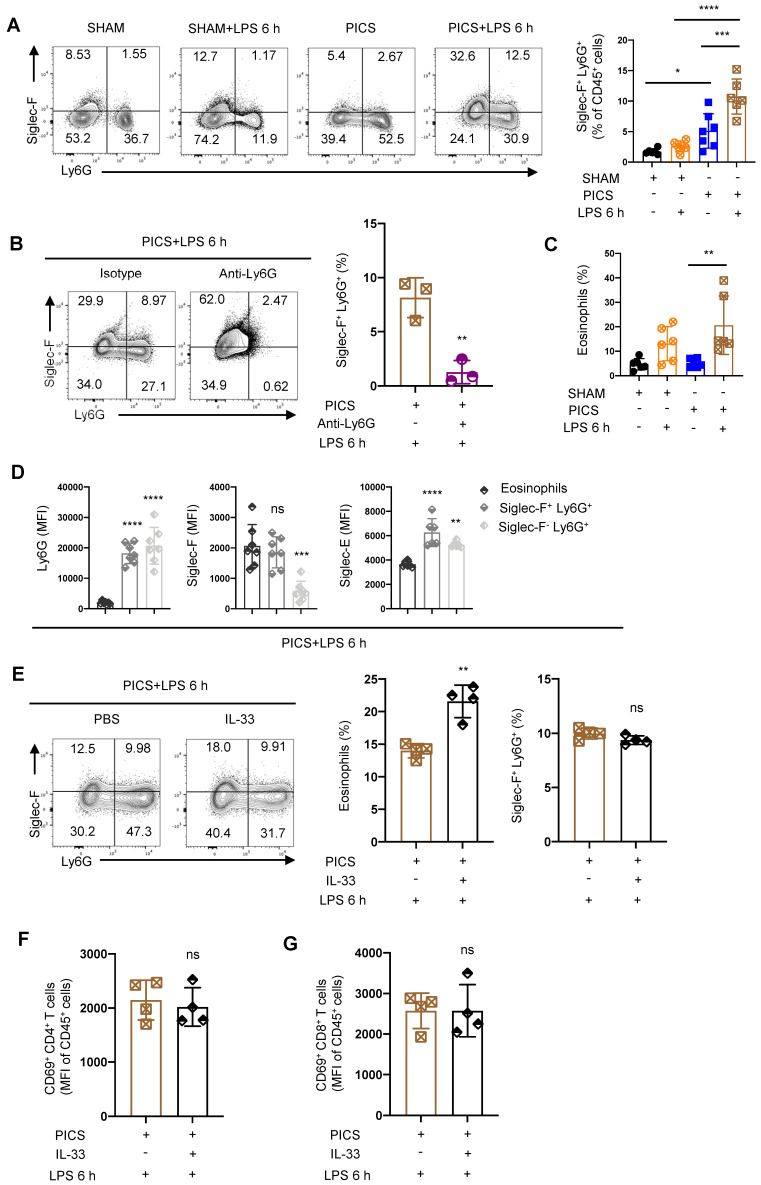
** Siglec-F was highly expressed in the splenic neutrophils of the PICS and PICS+LPS 6 h mice. (A)** Proportion of Siglec-F^+^ neutrophils in the spleens detected by flow cytometry. **(B)** After depletion of the neutrophils, proportion of Siglec-F^+^ neutrophils in the spleens of PICS+LPS 6 h and PICS+Anti-Ly6G+LPS 6 h mice examined by flow cytometry. **(C)** Proportion of the eosinophils detected by flow cytometry after secondary challenge with LPS. **(D)** Fluorescence intensity of Ly6G, Siglec-F and Siglec-E in eosinophils, Siglec-F^+^ neutrophils and Siglec-F^-^ neutrophils detected by flow cytometry. **(E-G)** After intraperitoneal injection IL-33 to expand eosinophils in PICS mice, proportion of eosinophils and Siglec-F^+^ neutrophils after secondary challenge, and activity of CD4^+^ and CD8^+^ T lymphocytes in the spleens examined by flow cytometry. One-way ANOVA followed by Dunnett's post hoc test was applied in **(A, C-D)**. Student's *t* test was applied in** (B, E-G)**. Data are presented as mean ± SEM. n = 3-6, *ns P* >0.05, *P <0.05, **P < 0.01, ***P < 0.001, ****P < 0.0001.

Siglec-F serves as a surface-specific marker for eosinophils, and Siglec-F^+^ neutrophils exhibit distinct functions in various environments 39. For example, Siglec-F^+^ neutrophils respond to DAMP signals, promote lung tumor development, participate in the cardiac repairment following the post-myocardial infarction, exacerbate air pollutant-induced airway inflammation, and protect nasal epithelium from inflammation caused by allergies and bacterial infections 40-49. Furthermore, depletion of neutrophils followed by LPS stimulation resulted in a significant decrease in the proportion of Siglec-F^+^ Ly6G^+^ cells (Figure 5B).

We also noted a significant increase in eosinophils in PICS+LPS 6 h mice (Figure 5C). To discern whether Siglec-F^+^ Ly6G^+^ cells are equivalent to Ly6G^+^ eosinophils, we performed surface characterization analysis. Both Siglec-F^+^ Ly6G^+^ cells and Siglec-F^-^ Ly6G^+^ cells displayed high expression of neutrophils biomarkers Siglec-E and Ly6G, while eosinophils uniquely exhibited high expression of Siglec-F (Figure 5D). This suggests that Siglec-F^+^ Ly6G^+^ cells are distinct from eosinophils, which highly express Ly6G.

To further distinguish between Siglec-F^+^ Ly6G^+^ cells and eosinophils, we administered recombinant IL-33 protein to PICS mice. Known to regulate the expansion of eosinophils 50. If Siglec-F^+^ Ly6G^+^ cells were eosinophils, IL-33 treatment would lead to their expansion. However, while IL-33 significantly increased the proportion of eosinophils, it did not alter the proportion of Siglec-F^+^ Ly6G^+^ cells (Figure 5E). Instead, the proportion of Siglec-F^-^ Ly6G^+^ cells, conventional neutrophils, and CD11b^+^ cells was downregulated (Figure S8B-D). This may result from a relative decrease in these cell populations due to the increased eosinophils, rather than an absolute decrease in their numbers. Moreover, IL-33 treatment did not affect the proportions or activity of CD45^+^ cells, CD3^+^, CD4^+^, or CD8^+^ T lymphocytes (Figure S8E-H, 5F-G).

### Unique Siglec-F^+^ neutrophils in the spleen of sepsis-induced immunosuppressed mice

**Figure 6:**
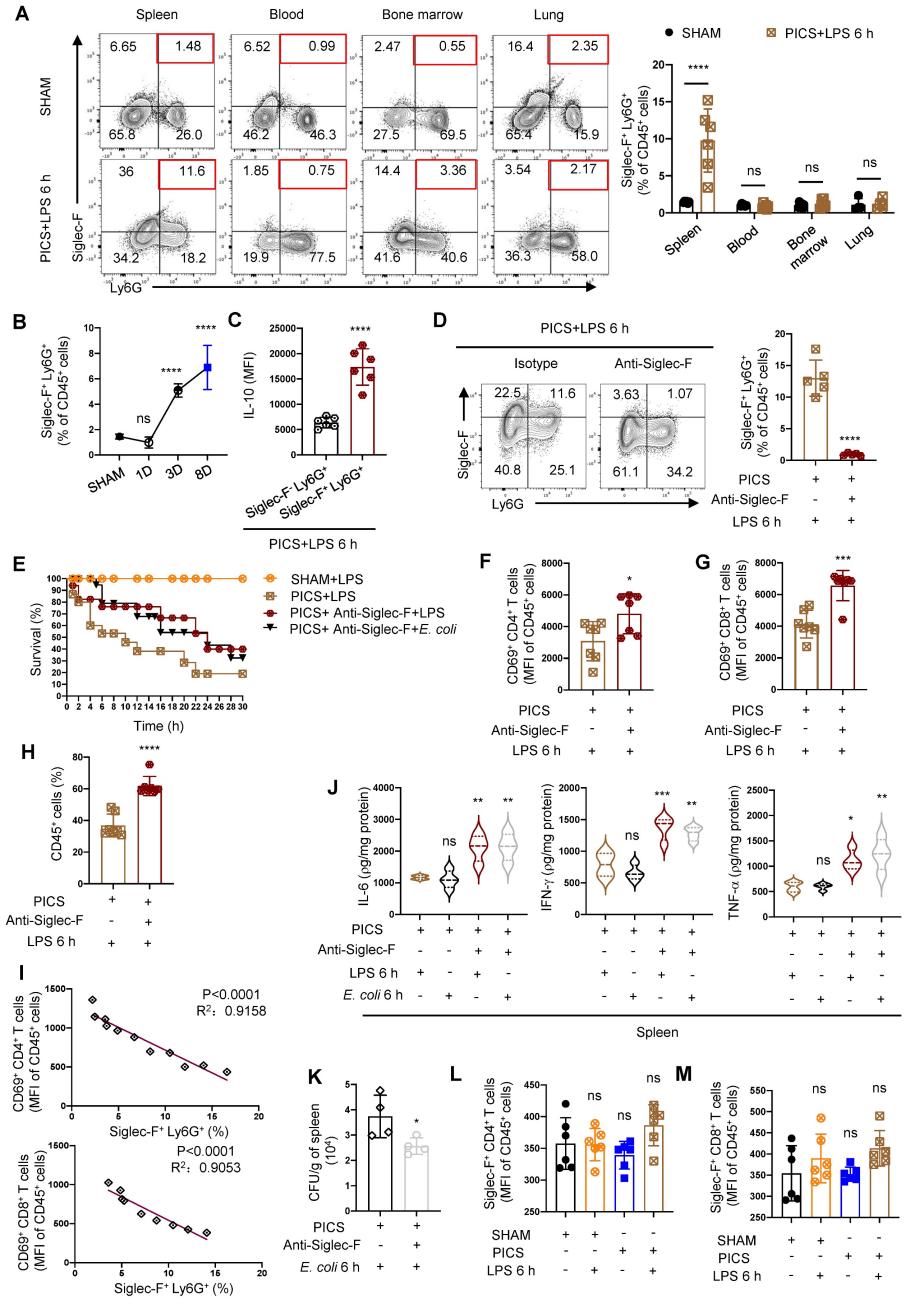
** Splenic neutrophils express Siglec-F specifically in PICS mice. (A)** Proportion of Siglec-F^+^ Ly6G^+^ in the spleen, peripheral blood, bone marrow and lung tissues of SHAM and PICS+LPS 6 h mice detected by flow cytometry. **(B)** Proportion of Siglec-F^+^ Ly6G^+^ in the spleens on SHAM and the first, third, eighth day after CLP detected by flow cytometry. **(C)** Expression of IL-10 in splenic Siglec-F^+^ Ly6G^+^ and Siglec-F^-^ Ly6G^+^ of PICS+LPS 6 h mice detected by flow cytometry. **(D, F-H)** After depleting Siglec-F in PICS mice, activity of CD4^+^ and CD8^+^ T lymphocytes and proportion of CD45^+^ Immune cells in the spleens after secondary challenge examined by flow cytometry. **(E)** PICS mice intraperitoneally injected with LPS or *E. coli* after treated with Anti-Siglec-F (n = 17), compared for survival with PICS+LPS mice (n = 15) and SHAM+LPS mice (n = 12). **(I)** Correlation between changes in proportions of Siglec-F^+^ neutrophils and activity of CD4^+^, CD8^+^ T lymphocytes demonstrated by correlation analysis. **(J)** Expression of IL-6, IFN-γ, TNF-α in the spleens of PICS mice and PICS+Anti-Siglec-F mice intraperitoneally injected LPS or *E. coli* examined by enzyme-linked immunosorbent assay. **(K)** Bacterial CFU per grams of spleen 6 h following *E. coli* infection. **(L-M)** Expression of Siglec-F in CD4^+^ and CD8^+^ T lymphocytes detected by flow cytometry. One-way ANOVA followed by Dunnett's post hoc test was applied in **(A-B, J-L)**. Student's *t* test was applied in** (C-D, F-H)**. Pearson's correlation test was applied in **(I)**. Data are presented as mean ± SEM. n = 4-17, *ns P* >0.05, *P <0.05, ***P < 0.001, ****P < 0.0001.

To uncover the origin of Siglec-F^+^ neutrophils during the immunosuppressive phase of sepsis, we investigated this population in the spleen, peripheral blood, bone marrow, and lung tissues of SHAM and PICS+LPS 6 h mice. Strikingly, Siglec-F^+^ neutrophils were predominantly expressed in the spleen and nearly absent in peripheral blood, bone marrow, and lung tissues, suggesting a unique localization of these cells in the spleen (Figure 6A).

The temporal dynamics of Siglec-F^+^ neutrophils production in the spleen and its association with the function and activity of T lymphocytes remained elusive. As sepsis progressed following CLP surgery, we observed a gradual increase in Siglec-F^+^ neutrophils, with significant differences detected on the third day post-surgery (Figure 6B). This increase correlated with decreased activity of CD4^+^, CD8^+^ T lymphocytes post-CLP surgery (Figure 3H-I). Moreover, a considerable proportion of Siglec-F^+^ neutrophils were found to secrete IL-10 (Figure S4A), with a higher expression in Siglec-F^+^ Ly6G^+^ cells compared Siglec-F^-^ Ly6G^+^ cells (Figure 6C), suggesting that IL-10-producing neutrophils were predominantly of the Siglec-F^+^ subtype.

To discern the function of Siglec-F^+^ neutrophils, we depleted Siglec-F in PICS mice (Figure 6D), resulting in significant activation of CD4^+^ and CD8^+^ T lymphocytes and improved survival under “second hit” (Figure 6E-G). Furthermore, the substantial increase in CD45^+^ cells indicated an enhanced immune response (Figure 6H). Importantly, a negative correlation was observed between Siglec-F^+^ neutrophils and the activity of CD4^+^ and CD8^+^ T lymphocytes (Figure 6I).

Considering that Siglec-F^+^ Ly6G^+^ cells are crucial in combating bacterial infection 45, we further investigated their role in secondary infection by stimulating mice with Escherichia coli (*E. coli*) during the immunosuppressive phase after depleting Siglec-F. Consistently, improved survival of PICS mice and increased production of proinflammatory cytokines were observed, indicating an enhanced immune response whether stimulated with *E. coli* or LPS (Figure 6E, J). And the depletion of Siglec-F increased host resistance to *E. coli* and reduced microbial load in the spleens during secondary infections (Figure 6K). Taking together, the removal of Siglec-F played a protective role during secondary challenge, which perhaps have involved other cellular interactions. Furthermore, we found no expression of Siglec-F on CD4^+^ and CD8^+^ T lymphocytes (Figure 6L-M), highlighting the specificity of Siglec-F expression on neutrophils.

In summary, during the immunosuppressive phase and subsequent secondary insult in septic mice, there was a distinct expression of Siglec-F in splenic neutrophils, which produced significant amounts of IL-10 and suppressed the function and activity of T lymphocytes.

### Siglec-F^+^ neutrophils suppress the function and activity of T lymphocytes in vitro

**Figure 7:**
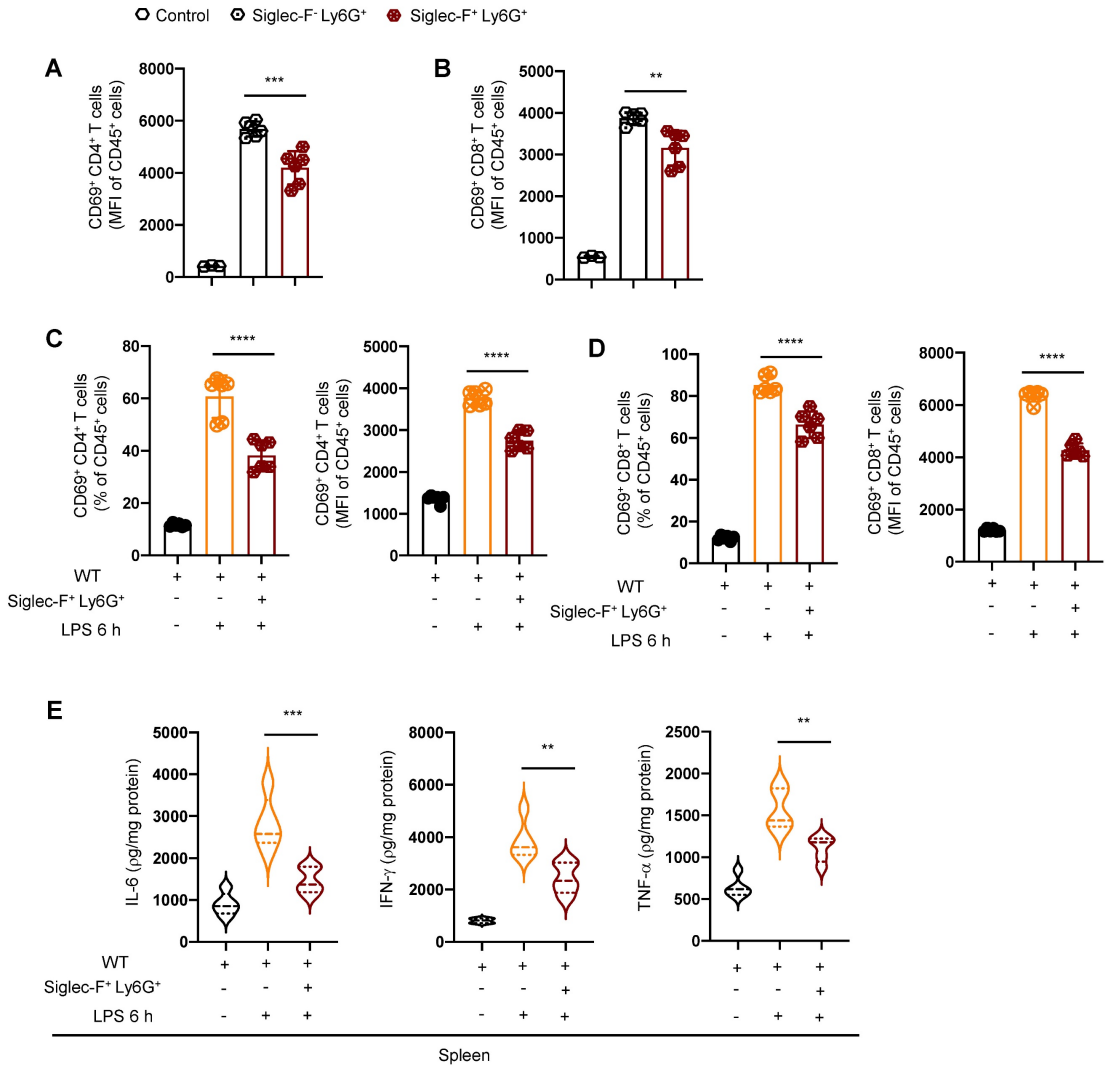
** Siglec-F^+^ neutrophils inhibit the activity of T lymphocyte in vitro. (A-B)** Petri dishes coated with Anti-CD3 (5 μg/ml) for 2 h, splenic T lymphocytes from PICS mice sorted by flow cytometry and co-cultured with Siglec-F^+^ Ly6G^+^ and Siglec-F^-^ Ly6G^+^ separately. Fluorescence intensity of CD69^+^ CD4^+^ and CD69^+^ CD8^+^ T lymphocytes detected by flow cytometry after stimulation with Anti-CD28 (5 μg/ml) for 6 h. **(C-E)** Adoptive transfer experiments: Siglec-F^+^ neutrophils (1x10^6^) from spleens of PICS mice transplanted into WT mice via the tail vein. Proportion and fluorescence intensity of CD69^+^ CD4^+^ and CD69^+^ CD8^+^ T lymphocytes detected by flow cytometry after 6 h stimulation with LPS. Enzyme-linked immunosorbent assay used to detect the expression of IL-6, IFN-γ, TNF-α in the spleens. One-way ANOVA followed by Dunnett's post hoc test was applied in **(A-E)**. Data are presented as mean ± SEM. n = 3-6, **P < 0.01, ***P < 0.001, ****P < 0.0001.

To further elucidate the capacity of Siglec-F^+^ neutrophils to suppress the function and activity of T lymphocytes, we conducted flow cytometry-based cell sorting of T lymphocytes from the spleen of PICS mice and co-cultured them with either Siglec-F^+^ Ly6G^+^ cells or Siglec-F^-^ Ly6G^+^ cells. Remarkably, T lymphocytes co-cultured with Siglec-F^-^ Ly6G^+^ cells displayed significantly enhanced activation induced by LPS compared to those co-cultured with Siglec-F^+^ Ly6G^+^ cells (Figure 7A-B).

Moreover, we performed adoptive transfer experiments by transplanting Siglec-F^+^ neutrophils from the spleen of PICS mice into the WT mice via the tail vein injection, followed by challenged with intraperitoneally LPS injection. Strikingly, compared to the WT+LPS 6 h mice, the activity of T lymphocytes in the spleen of WT+Siglec-F^+^ Ly6G^+^+LPS 6 h mice were notably suppressed (Figure 7C-D). Additionally, a significant decrease in IL-6, IFN-γ, and TNF-α levels was observed in the spleen of WT+Siglec-F^+^ Ly6G^+^+LPS 6 h mice (Figure 7E), indicating suppression of the immune response.

These findings underscore the role of Siglec-F^+^ neutrophils from the spleen of PICS mice in suppressing the activity of T lymphocytes and inhibiting the splenic immune response.

## Discussion

In this study, we observed a significant decrease in survival in PICS mice upon the secondary infection, accompanied by immune hypo-responsiveness in the spleen, characterized by reduced expression of pro-inflammatory cytokines (IL-6, IFN-γ, and TNF-α) and diminished functionality of T lymphocytes 15, 51, 52. We found that this immune hypo-responsiveness was mediated by IL-10, primarily produced by neutrophils infiltration in the spleen. Notably, depletion of neutrophils improved the T lymphocyte function and activity, increased survival rates, and revealed the critical role of neutrophils in immune suppression during secondary infections. Furthermore, we identified a subset of neutrophils expressing high levels of Siglec-F as the primary producers of IL-10, which significantly contributed to T lymphocyte suppression. Depletion of Siglec-F significantly led to enhanced survival during the secondary infections.

Traditionally, neutrophils have been primarily associated with acute inflammatory responses and pathogens clearance 18. However, recent studies have highlighted their heterogeneity and diverse functions in different microenvironments, including regulation of immune responses during infection, chronic inflammation, and cancer 20-22. As sepsis progressed, splenic Siglec-F^+^ neutrophils were gradually increased, while the activity of CD4^+^ and CD8^+^ T lymphocytes was decreased. Furthermore, a negative correlation between the proportion of Siglec-F^+^ neutrophils and activity of CD4^+^ and CD8^+^ T lymphocytes. Additionally, splenic Siglec-F^+^ neutrophils transplanted into the WT mice suppressed the functionality and activity of T lymphocytes in spleen. A previous study showed tumor-associated Siglec-F^+^ neutrophils possess immunosuppressive functions 41. However, the molecular mechanisms by which Siglec-F^+^ neutrophils exert immunosuppression in the secondary infections during late-stage sepsis remain unclear. Our various experimental data indicated that Siglec-F^+^ neutrophils secreted IL-10, thereby inhibiting the function and activity of T lymphocytes. To date, there is a relative lack of research on neutrophils in the immunosuppressive microenvironment of sepsis due to their high plasticity 19. Here, we investigated the function of neutrophils and identified Siglec-F^+^ neutrophils as potential targets for immunotherapy against the secondary infections. Our study provided novel insights into the immunosuppressive function of neutrophils in the microenvironment of the secondary infections.

One limitation of our study is the uncertainty regarding the systemic effects of neutrophil depletion, as our focus was primarily on the spleen. Future studies should explore the systemic impact of neutrophil depletion on other vital organs. Additionally, while we have identified IL-10 production by Siglec-F^+^ neutrophils as a mechanism of T lymphocyte suppression, further investigation is needed to elucidate other potential mechanisms, such as the release of reactive oxygen species or neutrophil extracellular traps (NETs), Furthermore, while our study has significant translational value, validation in clinical settings is warranted to confirm the relevance of our findings in patients.

In conclusion, our study highlights the detrimental role of Siglec-F^+^ neutrophils in the spleen-mediated immune suppression during late-stage sepsis. Targeting this subset of neutrophils may offer promising avenues for the treatment of secondary infections in sepsis patients. Further research is needed to fully understand the mechanisms underlying neutrophil-mediated immune suppression and to validate our findings in clinical settings.

## STAR Methods

### Mice

Male 6- to 8-week-old C57BL/6 mice were purchased from Guangdong Sja Biotechnology Co., Ltd. (license number: SCXK (yue) 2020-0052). The mice were housed in the Experimental Animal Center (SPF grade) of Affiliated Hospital of Guangdong Medical University Guangdong, China, under standardized conditions with a 12 h light/dark cycle and free access to food and water. All animal experimental protocols were approved by the Animal Ethics Committee of Affiliated Hospital of Guangdong Medical University.

### PICS mice models

Six- to eight-week-old C57BL/6 male mice was subjected to either underwent laparotomy with or without cecal ligation and perforation under surgical anesthesia with 2% sevoflurane and aseptic condition. Briefly, the distal cecum was ligated with silk thread at 1/3 of the distal cecum, and a 20 G artery indenture needle was used to puncture through the center of the distal cecum that had been ligated, and a small amount of feces was squeezed out, then the cecum was pushed back into the abdominal cavity, and the abdomen was closed with sutures. The feature of successful model as SHAM mice had rapidly response to stress and smooth hair; PICS mice had weight loss, slowly response to stimulation and movement, periocular and perianal purulent secretion.

### Murine splenic lymphocyte isolation

Spleens were ground and suspended in RPMI-1640 medium. Splenocyte suspensions were added with separation liquid (TBD Science, China) and centrifuged for 30 min, and the liquid was separated into four layers (from top to bottom: the diluent layer, ring-shaped milky white lymphocyte layer, transparent separating liquid layer, red blood cell layer). Erythrocytes were lysed with 2-3 ml of 1 × ACK lysis buffer for 3-5 min and lymphocytes were acquired for subsequent experiments.

### T lymphocyte isolation and culture

T lymphocytes were selectively collected from splenic lymphocytes with EasySep™ Mouse T Cell Isolation Kit (Cat^#^ 19851, Stemcell). 1 × 10^8^ splenic lymphocytes were added with 50 µl/ml of rat's serum and the isolation cocktail that incubated at room temperature for 10 min. The cells were mixed and incubated with vortexed RapidSpheres™. The suspensions were mixed by gently pipetting up and down 2-3 times and the tube (without lid) was placed into the magnet and incubated at room temperature for 2.5 min. T lymphocytes were ready and cultured in RPMI 1640 medium supplemented with 10% Fetal Bovine Serum (FBS) under standard conditions.

### Flow cytometric analysis, cell sorting, and subsequent analyses

Single mouse cell preparations from the spleen, peripheral blood, bone marrow and lung were resuspended in PBS and rat Anti-mouse CD16/32 and incubated for 10 min. For surface marker staining, cells were resuspended in FACS buffer (PBS with 2% FBS) and incubated for 30 min with the corresponding antibodies. For the intracellular staining assays, after fixation and permeabilization with BD Biosciences Cytofix/Cytoperm kit according to the manufacturer's instructions, the cells were incubated with antibodies. For the intranuclear staining assays, cells were fixed and permeabilized with eBioscience Foxp3/transcription factor staining kit according to the manufacturer's instructions. The cells were then incubated for an hour in Foxp3/transcription factor staining buffer with antibodies. Flow cytometry acquisition was performed on BD LSR II (BD) using FACSDiva software, and the data were processed with Flow Jo software. To obtain neutrophils of the PICS mice's spleen that did and did not express Siglec-F, cells were stained with the flow cytometric analysis antibodies. The cells were sorted as CD45^+^ CD11b^+^ Ly6G^+^, and Siglec-F positive or negative with the BD Biosciences FACSAria III cell sorter. Then the cells were co-cultured with T lymphocytes derived from the spleen of the PICS mice. Antibody are used as indicated in key resources table S1.

### Anti-IL-10R Ab treatment

To test the role of IL-10 in the spleen, mice were treated with 200 μg of Anti-IL-10R Ab (clone 1B1.3A, BioXCell) or a control rat IgG1 Ab (clone HRPN, BioXCell) on day 4 and 6 after surgery.

### Inhibiting neutrophil recruitment

CXCR2 antagonist SB225002 was suspended in the special solvent that consisted of 30% polyethylene glycol, 5% Tween-80, 2% dimethyl sulfoxide, and 63% water. The PICS mice were injected with SB225002 at 10 mg/kg i.p. on postoperative day 4, 5, 6 and 7.

### Siglec-F^+^ neutrophil depletion with Anti-Siglec-F and Anti-Ly6G antibodies

To test the role of Siglec-F^+^ neutrophils in the spleen, PICS mice were depleted of these cells by injecting Siglec-F or Ly6G depletion antibodies. On days 4, 5, 6 and 7 after surgery, mice were i.p. injected with the isotype antibody (40 μg/200 μl PBS; KLH, R&D Systems), the Siglec-F depletion antibody (40 μg/200 μl PBS; Q920G3, R&D Systems), or the Ly6G depletion antibody (250 μg/200 μl; 1A8, BioXCell). Flow cytometric analysis was performed to evaluate the degree of depletion by the treatments.

### Macrophage depletion with liposomes

To test the role of macrophage in the spleen, macrophage of the PICS mice was depleted with injecting liposomes on days 7 after surgery (200 μl at 5 mg/ml i.p).

### Treg depletion with Anti-IL-2 antibody

To test the role of Treg in the spleen, Treg in the PICS mice were depleted with injecting Treg depletion antibody. On days 4, 5, 6 and 7 after surgery, mice were i.p. injected with the isotype antibody (150 μg/200 μl PBS; 2A3, BioXCell) or the Treg depletion antibody (150 μg/200 μl; S4B6-1, BioXCell).

### Expansion of eosinophil populations with IL-33 injections

To test whether the Siglec-F^+^ neutrophils are eosinophils, the PICS mice were i.p. injected with 250 ng recombinant IL-33 (Chamot) on postsurgical day 4, 5, 6 and 7.

### Adoptive transfer of Siglec-F^+^ neutrophils

Siglec-F^+^ neutrophils were prepared from the PICS mice's spleen by Flow cytometric sorting. WT mice were i.v. injected with or without 1 × 10^6^ cells of Siglec-F^+^ neutrophils and then i.p. with LPS 24 h later. All mice were euthanized 6 h after LPS injection and investigated for CD4^+^, CD8^+^ T lymphocyte activity and proinflammatory mediators in the spleen using Flow cytometric and qPCR analysis.

### *E. coli* infection

A single colony of bacteria grown on LB agar plates containing kanamycin (50 μg/ml) were placed in 10 ml LB broth containing kanamycin and grown statically overnight in a bacterial vibrator. Bacteria were prepared at 5 × 10^8^ CFU/ml in PBS, each mouse was intraperitoneally injected with 200 μl for an infectious dose of 1 × 10^8^ CFU/mouse.

### Microbial counts

Mice infected with *E. coli* were euthanized 6 h after infection. The spleen was collected and homogenized with a PowerGen 125 homogenizer (Fisher Scientific) for 30 s, and the diluent of the spleen were plated to an LB agar plates to determine the CFU.

### Quantitative real-time RT-PCR

Total RNA purified from spleen, lung, heart and liver was extracted using an TRIzol™ Plus RNA Purification Kit (Invitrogen) and reverse-transcribed using TaKaRa reverse transcriptase. qPCR was performed using performed using TB Green® Premix Ex Taq™ II (TaKaRa) on a Light Cycler 480 (Roche) instrument. Data were analyzed with the comparative Ct (2^-ΔΔCt^) method relative to the housekeeping genes as indicated in the figures. Primers are used as indicated in key resources table S2.

### RNA-seq

Total RNA was isolated from spleen using Trizol reagent. Then mRNA samples library construction and high-throughput sequencing were performed by Hangzhou KaiTai Biotechnology Co., Ltd. In brief, Polyadenylated RNA was enriched from total RNA. mRNA samples were fragmented into 300-nucleotide-long fragments using RNA Fragmentation Reagents (Ambion, AM8740) and used for library construction and performed high-throughput sequencing on Illumina Novaseq 6000.

### Histological analysis

Tissues were formalin-fixed, processed, and paraffin-embedded, and stained with hematoxylin and eosin (H&E) (Jiangsu Meimian Industrial Co., Ltd, Jiangsu, China).

### Data and code availability

RNA-seq raw data are available in the Gene Expression Omnibus database (https://www.ncbi.nlm.nih.gov/geo/) under accession number GSE264139.

### Ethics statement

All experiments involving animals were conducted according to the ethical policies and procedures approved by the Animal Ethics Committee of Affiliated Hospital of Guangdong Medical University.

### Statistical analysis

The data are presented as mean ± SEM. Differences between two groups were analyzed by Student's *t* test. Multi comparisons were compared using one-way ANOVA followed by Dunnett's post hoc test. Correlation analyses were conducted with Pearson's correlation test. Statistical analysis was performed with GraphPad Prism 9 software (GraphPad software, CA, USA). A *p* value less than 0.05 was considered to be statistically significant.

## Supplementary Material

Supplementary figures and table.
