# Supplementary material for: Siglec-F+ neutrophils in the spleen induce immunosuppression following acute infection
Source: Theranostics. 2024 Apr 8;14(6):2589–604. doi: 10.7150/thno.93812 (PMC11024851; doi:10.7150/thno.93812)
Supplement: Supplementary file 1 — Supplementary figures and table. [file thnov14p2589s1.pdf]

Key resources table S1

REAGENT or RESOURCE	SOURCE	IDENTIFIER
Antibodies		
Purified anti-mouse CD16/32	Biolegend	101302
LIVE/DEAD™ Fixable Red Dead Cell Stain	Biolegend	423110
7-AAD Viability Staining Solution	Biolegend	420404
PE/Cyanine7 anti-mouse CD45	Biolegend	103114
PerCP/Cyanine5.5 anti-mouse CD45 Antibody	Biolegend	157208
APC/Cyanine7 anti-mouse CD45 Antibody	Biolegend	157618
Brilliant Viloet 605™ anti-mouse CD45 Antibody	Biolegend	103140
APC anti-mouse Ly-6G Antibody	Biolegend	127614
APC anti-mouse/human CD11b Antibody	Biolegend	101212
FITC anti-mouse CD170 (Siglec-F) Antibody	Biolegend	155504
PE anti-mouse Siglec-E Antibody	Biolegend	677103
APC anti-mouse CD182 (CXCR2) Antibody	Biolegend	149312
FITC Rat IgG2a, κ Isotype Ctrl Antibody	Biolegend	400505
PE anti-mouse Ly-6G Antibody	Biolegend	127608
PE/Cyanine7 anti-mouse/human CD11b Antibody	Biolegend	101216
FITC anti-mouse F4/80 Antibody	Biolegend	123108
PE anti-mouse NK-1.1 Antibody	Biolegend	156504
PE/Cyanine7 anti-mouse CD31 Antibody	Biolegend	102418

APC anti-mouse Ly-6C Antibody	Biolegend	128016
APC anti-mouse CD19 Antibody	Biolegend	152410
APC/Cyanine7 anti-mouse CD3 Antibody	Biolegend	100222
APC anti-mouse CD8b.2 Antibody	Biolegend	140410
Brilliant Viloet 510™ anti-mouse CD8b(Ly-3) Antibody	Biolegend	126631
PE anti-mouse CD25 Antibody	Biolegend	101903
FITC anti-mouse CD69 Antibody	Biolegend	104506
FITC anti-mouse CD62L Antibody	Biolegend	161212
PE anti-mouse CD274(B7-H, PD-L1) Antibody	Biolegend	124308
APC anti-mouse CD4 Antibody	Biolegend	100412
PE anti-mouse CD4 Antibody	Biolegend	100408
Brilliant Viloet 510™ anti-mouse CD3 Antibody	Biolegend	100234
Brilliant Viloet 421™ anti-mouse FOXP3 Antibody	Biolegend	126419
PerCP/Cyanine5.5 anti-house/mouse/rat CD278(ICOS)Antibody	Biolegend	313518
APC anti-mouse IFN-γ Antibody	Biolegend	505810
PerCP/Cyanine5.5 anti-mouse IL-4 Antibody	Biolegend	504124
APC anti-mouse IL-17A Antibody	Biolegend	506916
Brilliant Viloet 421™ anti-mouse IL-10 Antibody	Biolegend	505022
Chemicals, peptides, and recombinant proteins		
Lipopolysaccharide	Sigma-Aldrich	L2880

Clophosome®-A-Clodronate Liposomes (Anionic)	FormuMax	F70101C-A
SB225002	Selleck	S7651
Mouse Siglec-F Antibody	R&D	MAB17061-100
Mouse IgG1 Isotype Control	R&D	MAB002
InVivoPlus anti-mouse IL-10R (CD210)	BioXCell	BP0050
InVivoPlus rat IgG1 isotype control, anti-horseradish peroxidase	BioXCell	BP0088
InVivoPlus anti-mouse Ly6G	BioXCell	BP0075-1
InVivoPlus rat IgG2a isotype control, anti-trinitrophenol	BioXCell	BP0089
InVivoMAb anti-mouse IL-2	BioXCell	BE0043-1
InVivoMAb rat IgG2a isotype control, anti-trinitrophenol	BioXCell	BE0089
Recombinant IL-33	Chamot	CM030-MP
Critical commercial assays		
Mouse CXCL1/KC Quantikine ELISA Kit	R&D	MKC00B-1
Mouse CXCL2/MIP-2 Quantikine ELISA Kit	R&D	MM200
Mouse IFN-gamma Quantikine ELISA Kit	R&D	MIF00-1
Mouse IL-10 Quantikine ELISA Kit	R&D	M1000B-1
EasySep^{TM} Mouse T Cell I solution Kit	Stemcell	19851
Bacterial strains		
Escherichia coli	BioVector NTCC	ACCC01634

Key resources table S2

Primer list for the qPCR experiments.

Gene(s)	RT-qPCR Oligonucleotides
m-GAPDH-F	AGGTCGGTGTGAACGGATTTG
m-GAPDH-R	TGTAGACCATGTAGTTGAGGTCA
m-IL-6-F	TAGTCCTTCCTACCCCAATTTCC
m-IL-6-R	TTGGTCCTTAGCCACTCCTTC
m-IFN-γ-F	ATGAACGCTACACACTGCATC
m- IFN-γ-R	CCATCCTTTTGCCAGTTCCTC
m-TNFα-F	AAGCCTGTAGCCCACGTCGTA
m-TNFα-R	GGCACCACTAGTTGGTTGTCTTTG
m-IL-10-F	GGTTGCCAAGCCTTATCGGA
m-IL-10-R	GGGGAGAAATCGATGACAGC
m-CXCL1-F	CTGGGATTCACCTCAAGAACATC
m-CXCL1-R	CAGGGTCAAGGCAAGCCTC
m-CXCL2-F	CCAACCACCAGGCTACAGG
m-CXCL2-R	GCGTCACACTCAAGCTCTG
m-CXCL3-F	AGTGTGGCTATGACTTCGG
m-CXCL3-R	GAATTCACCTCAAGAACATCCA
m-CXCL5-F	TCCAGCTCGCCATTCATGC
m-CXCL5-R	TTGCGGCTATGACTGAGGAAG
m-CXCL8-F	TAAGTTCTTTAGCACTCCTTGG
m-CXCL8-R	TTCCTGATTTCTGCAGCTC

Figure S1 Indicators in the PICS mice model.

The PICS mice model was characterized by various indicators on the eighth day after cecal ligation and perforation (CLP).

(A-B) Proportion of macrophages and neutrophils in the spleens of PICS mice measured by flow cytometry.

(C-F) Proportion of neutrophils in the bone marrow and peripheral blood, and proportion of $CD4^+$ and $CD8^+$ T lymphocytes in the spleens of PICS mice assessed by flow cytometry.

(G-I) Fluorescence intensity of PD-$L1^+$ $CD4^+$ T lymphocyte, PD-$L1^+$ $CD8^+$ T lymphocyte, and $FoxP3^+$ $CD4^+$ T lymphocyte in the spleens, as well as proportion of $FoxP3^+$ $CD4^+$ T lymphocytes, determined by flow cytometry.

(J) Expression of IL-10 in splenic $CD45^+$ immune cells of PICS mice detected by flow cytometry.

(**K)** Percentage calculated by subtracting the preoperative weight of mice from the weight at day 8 after CLP or sham surgery, then dividing by the preoperative weight of mice.

All indicators of the PICS group compared with the SHAM group and statistically analyzed by Student's *t* test. Data are presented as mean ± SEM. n = 4-10, $*P < 0.05$, $**P < 0.01$, $***P < 0.001$, $****P < 0.0001$.

Figure S2 Proportions of immune cells in the spleens.

(A) Flow cytometry used to detect the proportion of lymphocytes, monocytes, and granulocytes in the spleens of the mice.

(B) Flow cytometry used to detect the proportion of $CD11b^+$ immune cells in the spleen, peripheral blood, and bone marrow of the mice.

(C-J) Flow cytometry used to detect the proportion of B cells, NK cells, naive $CD4^+$, $CD8^+$ T lymphocytes, Th1, Th2, Th17, and immature macrophages in the spleens of the mice. The results in the PICS and PICS+LPS 6 h groups were compared with the SHAM and SHAM+LPS 6 h groups, respectively, by one-way ANOVA followed by Dunnett's post hoc test. Data are presented as mean

± SEM. n = 3-10, *ns P* >0.05, **P* <0.05, ***P* < 0.01, ****P* < 0.001, *****P* < 0.0001.

Figure S3 Function of Treg cells in the spleen of PICS mice after secondary challenge.

(A-B) Proportions of Treg cells, $ICOS^+$ Treg, and fluorescence intensity of $ICOS^+$ Treg in the spleens were examined by flow cytometry.

(C) Efficiency of depleted Treg cells in the spleen of PICS mice.

(D-F) After Treg cells depletion, proportion of $CD45^+$ immune cells and expression of IL-10, IL-6, IFN-γ, and TNF-α in the spleens of PICS+LPS 6 h and PICS+Anti-IL-10R+LPS 6 h mice were examined by flow cytometry and enzyme-linked immunosorbent assay.

(G-H) Proportion of $CD4^+$ and $CD8^+$ T lymphocytes in $IL\text{-}10^+$ $CD45^+$ immune cells and fluorescence intensity of $TGF\beta^+$ $CD45^+$ immune cells were detected by flow cytometry.

One-way ANOVA followed by Dunnett's post hoc test was applied in **(A-B, G-H)**. Student's *t* test was applied in **(D-F)**. Data are presented as mean ± SEM. n = 3-7, *ns P* >0.05, **P* <0.05, ***P* < 0.01, ****P* < 0.001.

Figure S4 Expression of IL-10 in immune cells of the spleen.

(A) Expression of IL-10 in splenic neutrophils detected by flow cytometry.

(B) Relative mRNA expression of IL-6, IFN-γ, and TNF-α in splenic neutrophils of PICS mice and PICS+LPS 6 h mice examined by qPCR.

(C) Efficiency of depleting neutrophils in PICS mice.

(D) Apparent changes observed in the spleen after depleting neutrophils in PICS mice.

(E-G) Expression of IL-10 in NK cells, $CD3^+$, and $CD4^+$ T lymphocytes of the spleens examined by flow cytometry.

One-way ANOVA followed by Dunnett's post hoc test was applied in **(E-G)**. Student's *t* test was applied in **(B)**. Data are presented as mean ± SEM. n = 3-6, *ns P* >0.05, **P* <0.05, ***P* < 0.01.

Figure S5 Effects of depleting neutrophils on the spleen.

(A-E) Expression of IL-10 in the spleen and proportions of $CD45^+$ immune cells, $CD3^+$, $CD4^+$, and $CD8^+$ T lymphocytes after neutrophils depletion.

(F-H) Splenic neutrophils and T lymphocytes from PICS mice were sorted by flow cytometry. Neutrophils were stimulated with LPS (1 μg/ml) in vitro for 6 h, and the supernatants were co-cultured with the T lymphocytes pre-incubated with Isotype or Anti-IL-10R for 6 h. Subsequently, T lymphocytes were stimulated with LPS for 6 h, and their function and activity were detected by flow cytometry. The relative mRNA expression of IFN-γ in T lymphocytes was examined by qPCR.

Student's *t* test was applied in **(A-H)**. Data are presented as mean ± SEM. n = 3-7, *ns P* >0.05, **P* <0.05, ***P* < 0.01, ****P* < 0.001, *****P* < 0.0001.

Figure S6 Impact of the macrophages on T lymphocytes in the spleen.

(A-B) Proportion of splenic macrophages and expression of IL-10 in the splenic macrophages after secondary challenge, examined by flow cytometry.

(C) Relative mRNA expression of IL-10 in splenic macrophages detected by qPCR.

(D) Efficiency of macrophage depletion in PICS mice.

(E-G) After macrophage depletion, proportion of $CD45^+$ immune cells in PICS+LPS 6 h mice and PICS+Liposomes+LPS 6 h mice, and the activity of $CD4^+$ and $CD8^+$ T lymphocytes in the spleens were detected by flow cytometry.

One-way ANOVA followed by Dunnett's post hoc test was applied in **(A-C, F-G)**. Student's *t* test was applied in **(E)**. Data are presented as mean ± SEM. n = 3-6, *ns P* >0.05, **P* <0.05, ***P* < 0.01.

Figure S7 Expression of CXCLs in the spleen, T lymphocytes and macrophages.

(A-E) Expression of CXCL1, CXCL2, CXCL3, CXCL5, and CXCL8 in splenic T lymphocytes of SHAM, PICS, and PICS+LPS 6 h mice.

(F-I) mRNA expression of CXCL1, CXCL2, CXCL3, and CXCL5 in the spleens

of SHAM, PICS, and PICS+LPS 6 h mice, detected by qPCR.

(J) mRNA expression of CXCL2 in splenic macrophages of SHAM, PICS, and PICS+LPS 6 h mice, detected by qPCR.

One-way ANOVA followed by Dunnett's post hoc test was applied in **(A-J)**. Data are presented as mean ± SEM. n = 3-6, *ns P* >0.05, **P* <0.05, ***P* < 0.01, *****P* < 0.0001

Figure S8 Effects on immune cells with expanded eosinophils after secondary challenge.

(A) Proportion of Siglec-F^{-} neutrophils after secondary challenge detected by flow cytometry.

(B-H) After intraperitoneally injecting IL-33 to expand eosinophils of in PICS mice, the proportions of Siglec-F^{-} neutrophils, neutrophils, CD11b^{+} cells, CD45^{+} immune cells, CD3^{+}, CD4^{+}, and CD8^{+} T lymphocytes in PICS+LPS 6 h mice and in PICS+IL-33+LPS 6 h mice were examined by flow cytometry.

One-way ANOVA followed by Dunnett's post hoc test was applied in **(A)**. Student's *t* test was applied in **(B-H)**. Data are presented as mean ± SEM. n = 4-6, *ns P* >0.05, **P* <0.05, ***P* < 0.01, ****P* < 0.001.

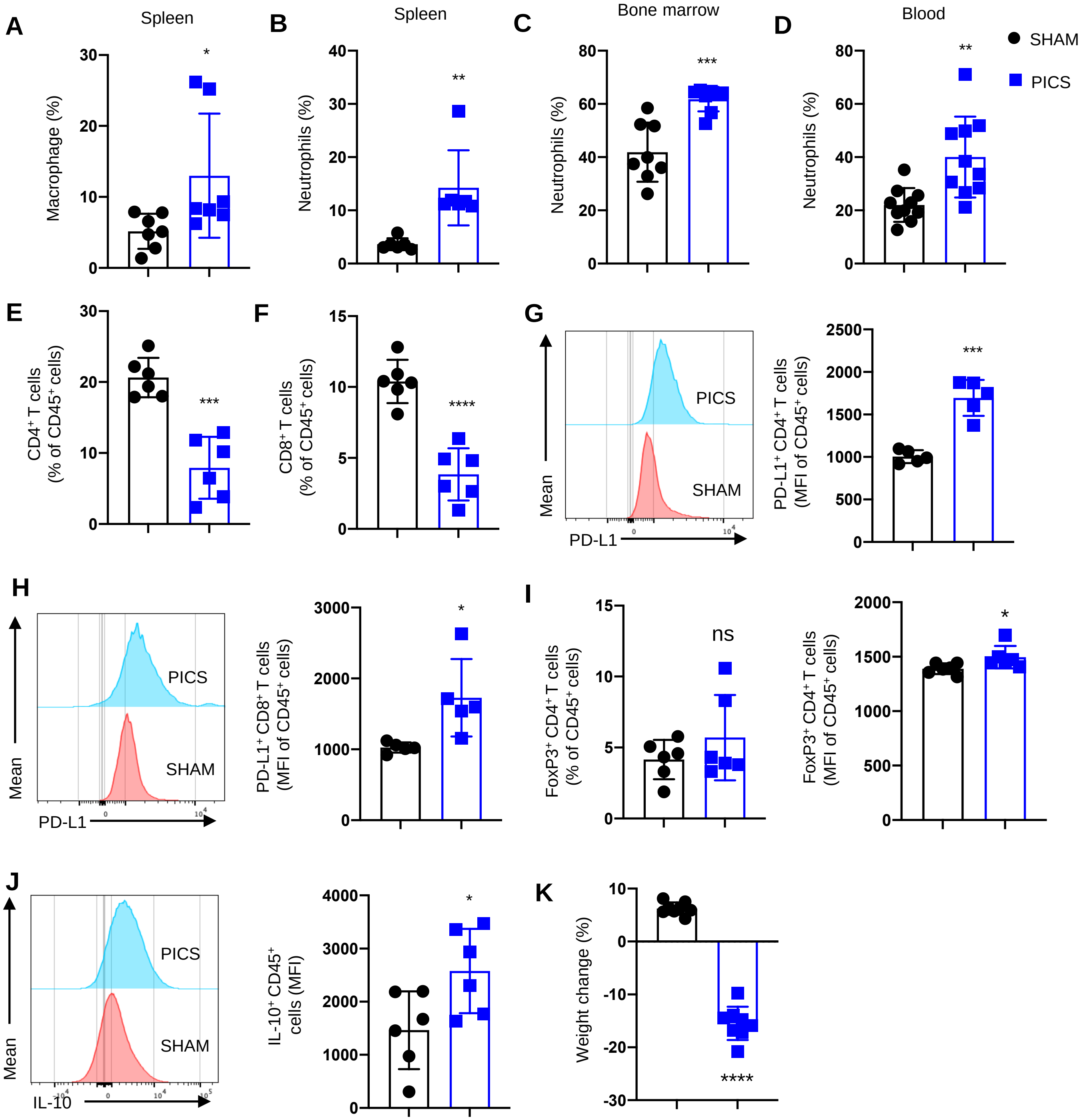

A
Spleen
Macrophage (%)
B
Spleen
Neutrophils (%)
C
Bone marrow
Neutrophils (%)
D
Blood
Neutrophils (%)
SHAM
PICS
E
CD4+ T cells (% of CD45+ cells)
F
CD8+ T cells (% of CD45+ cells)
G
PICS
SHAM
Mean
PD-L1
PD-L1+ CD4+ T cells (MFI of CD45+ cells)
H
PICS
SHAM
Mean
PD-L1
PD-L1+ CD8+ T cells (MFI of CD45+ cells)
I
FoxP3+ CD4+ T cells (% of CD45+ cells)
ns
FoxP3+ CD4+ T cells (MFI of CD45+ cells)
J
PICS
SHAM
Mean
IL-10
IL-10+ CD45+ cells (MFI)
K
Weight change (%)

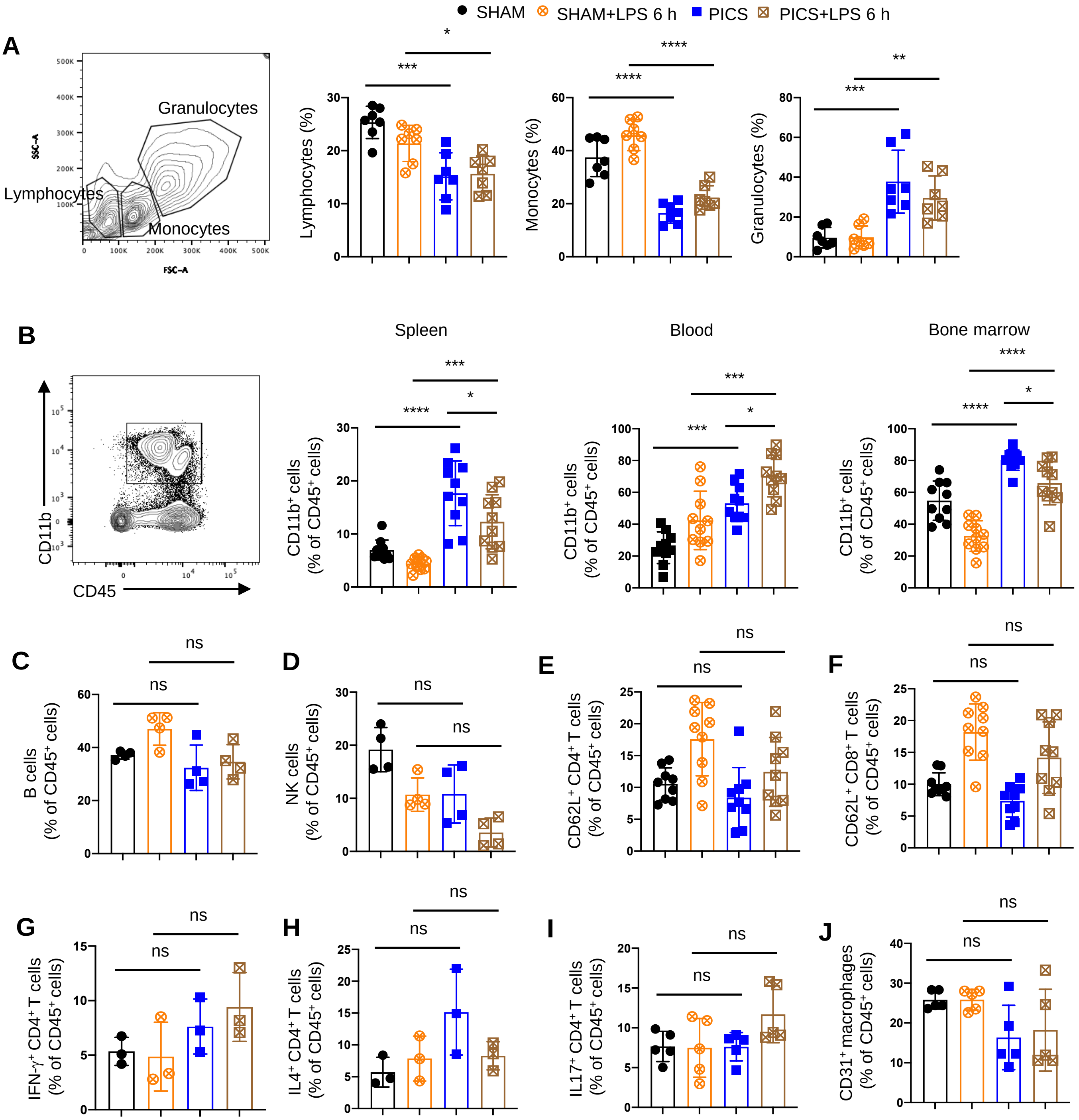

SHAM
SHAM+LPS 6 h
PICS
PICS+LPS 6 h
A
Granulocytes
Lymphocytes
Monocytes
SSC-A
FSC-A
Lymphocytes (%)
Monocytes (%)
Granulocytes (%)
B
Spleen
Blood
Bone marrow
CD11b
CD45
CD11b⁺ cells (% of CD45⁺ cells)
C
B cells (% of CD45⁺ cells)
D
NK cells (% of CD45⁺ cells)
E
CD62L⁺ CD4⁺ T cells (% of CD45⁺ cells)
F
CD62L⁺ CD8⁺ T cells (% of CD45⁺ cells)
G
IFN-γ⁺ CD4⁺ T cells (% of CD45⁺ cells)
H
IL4⁺ CD4⁺ T cells (% of CD45⁺ cells)
I
IL17⁺ CD4⁺ T cells (% of CD45⁺ cells)
J
CD31⁺ macrophages (% of CD45⁺ cells)
ns

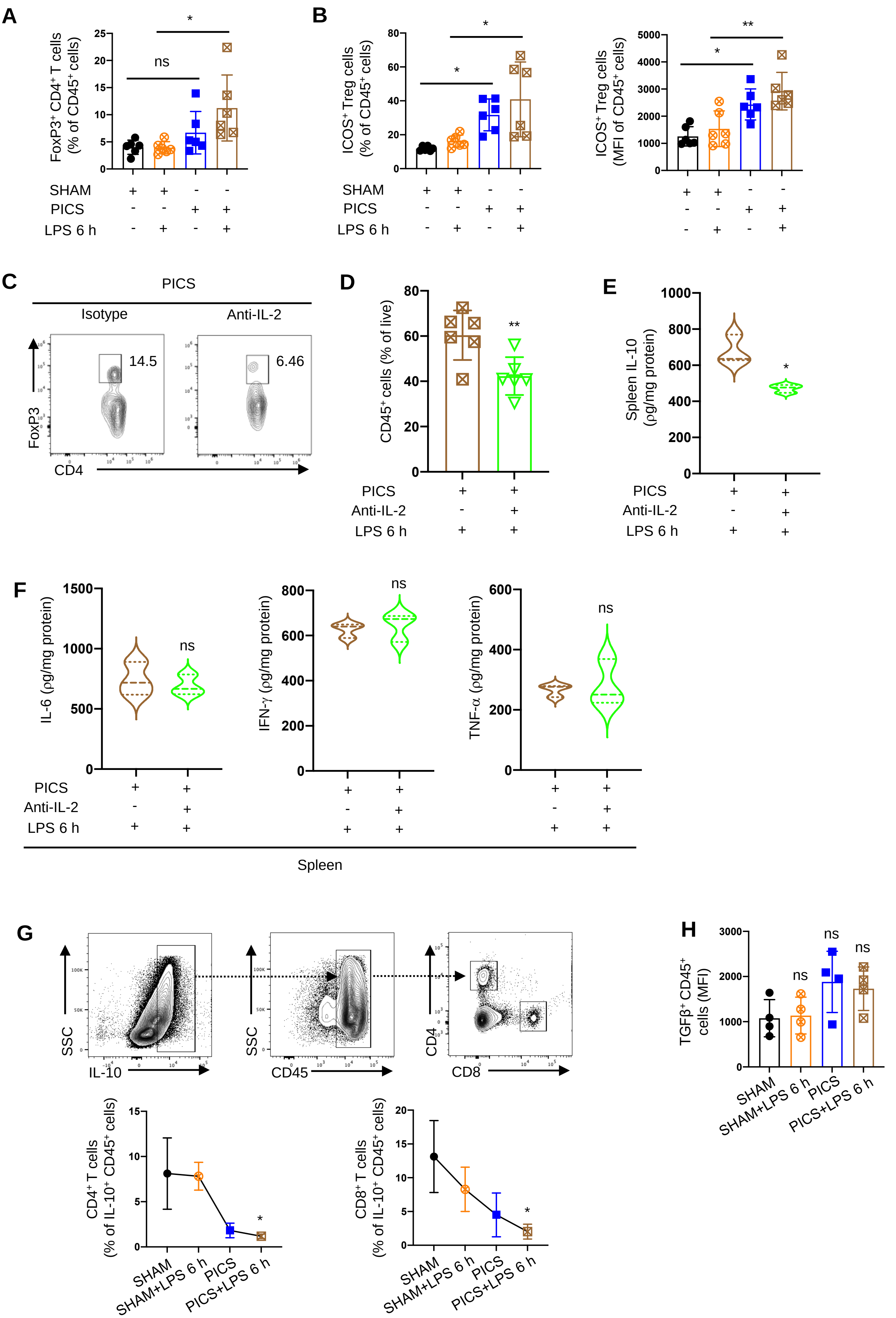
A
FoxP3⁺ CD4⁺ T cells (% of CD45⁺ cells)
ns
*
SHAM
PICS
LPS 6 h
B
ICOS⁺ Treg cells (% of CD45⁺ cells)
ICOS⁺ Treg cells (MFI of CD45⁺ cells)
*
**
C
PICS
Isotype
Anti-IL-2
14.5
6.46
FoxP3
CD4
D
CD45⁺ cells (% of live)
**
Anti-IL-2
E
Spleen IL-10 (ρg/mg protein)
*
F
IL-6 (ρg/mg protein)
IFN-γ (ρg/mg protein)
TNF-α (ρg/mg protein)
ns
Spleen
G
SSC
IL-10
CD45
CD4
CD8
CD4⁺ T cells (% of IL-10⁺ CD45⁺ cells)
CD8⁺ T cells (% of IL-10⁺ CD45⁺ cells)
SHAM
SHAM+LPS 6 h
PICS
PICS+LPS 6 h
H
TGFβ⁺ CD45⁺ cells (MFI)

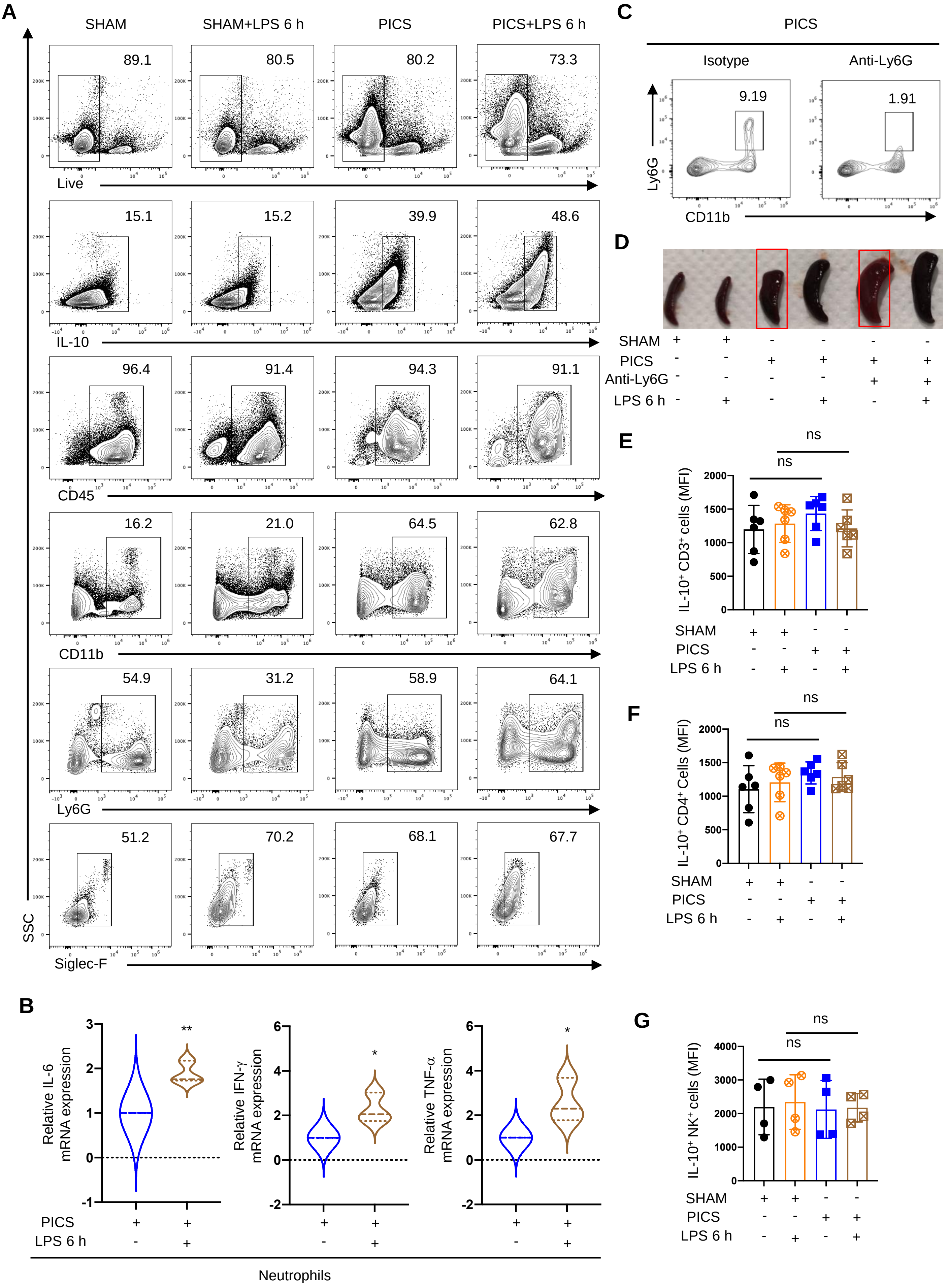
A
SHAM
SHAM+LPS 6 h
PICS
PICS+LPS 6 h
89.1
80.5
80.2
73.3
Live
15.1
15.2
39.9
48.6
IL-10
96.4
91.4
94.3
91.1
CD45
16.2
21.0
64.5
62.8
CD11b
54.9
31.2
58.9
64.1
Ly6G
51.2
70.2
68.1
67.7
Siglec-F
SSC
B
Relative IL-6 mRNA expression
Relative IFN-γ mRNA expression
Relative TNF-α mRNA expression
**
*
*
PICS
LPS 6 h
Neutrophils
C
PICS
Isotype
Anti-Ly6G
9.19
1.91
Ly6G
CD11b
D
SHAM
PICS
Anti-Ly6G
LPS 6 h
E
IL-10+ CD3+ cells (MFI)
ns
ns
F
IL-10+ CD4+ Cells (MFI)
G
IL-10+ NK+ cells (MFI)

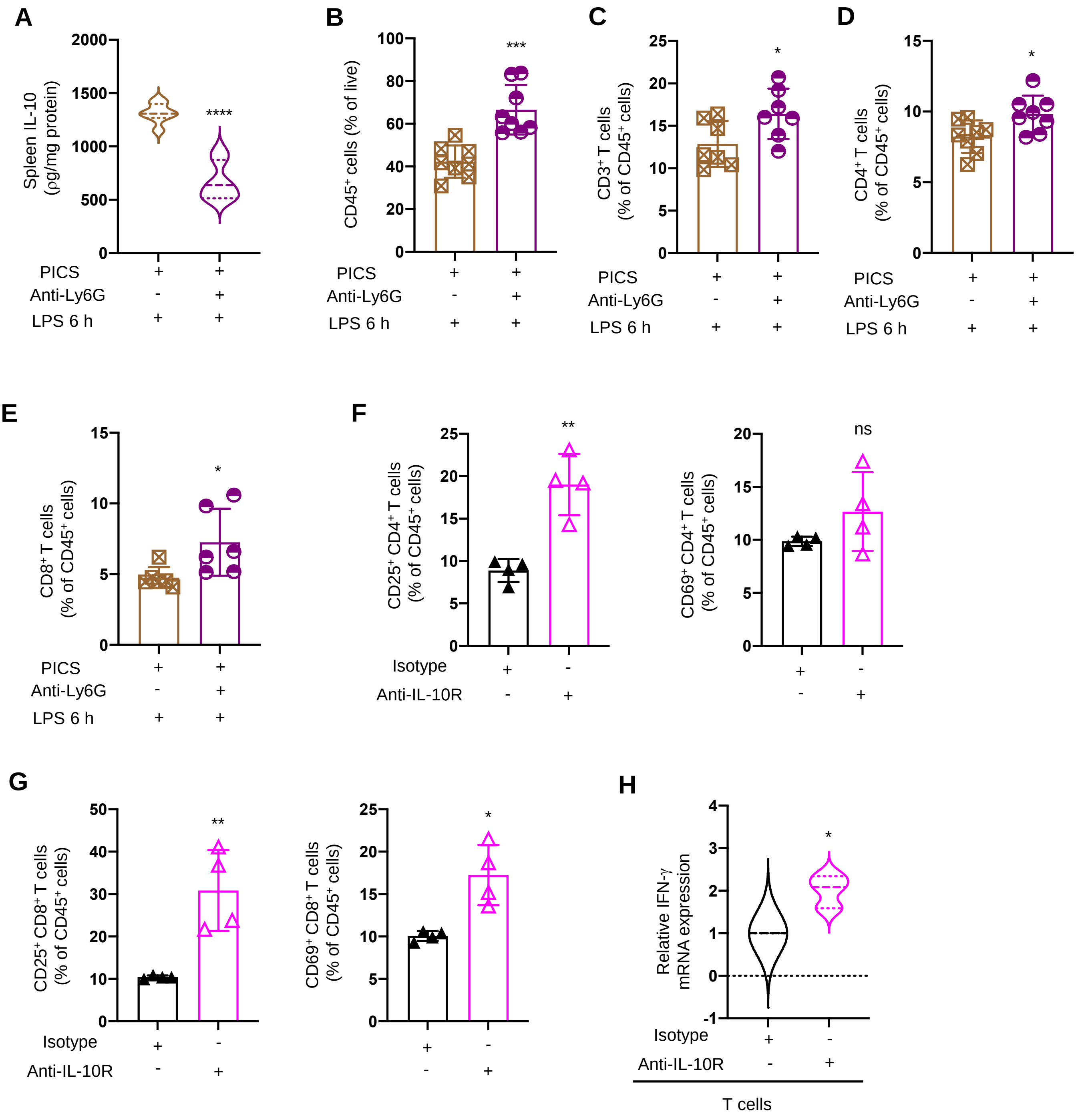
A
Spleen IL-10 (ρg/mg protein)
2000
1500
1000
500
0
****
PICS + +
Anti-Ly6G - +
LPS 6 h + +
B
CD45$^+$ cells (% of live)
***
PICS + +
Anti-Ly6G - +
LPS 6 h + +
C
CD3$^+$ T cells (% of CD45$^+$ cells)
*
PICS + +
Anti-Ly6G - +
LPS 6 h + +
D
CD4$^+$ T cells (% of CD45$^+$ cells)
*
PICS + +
Anti-Ly6G - +
LPS 6 h + +
E
CD8$^+$ T cells (% of CD45$^+$ cells)
*
PICS + +
Anti-Ly6G - +
LPS 6 h + +
F
CD25$^+$ CD4$^+$ T cells (% of CD45$^+$ cells)
**
CD69$^+$ CD4$^+$ T cells (% of CD45$^+$ cells)
ns
Isotype + -
Anti-IL-10R - +
G
CD25$^+$ CD8$^+$ T cells (% of CD45$^+$ cells)
**
CD69$^+$ CD8$^+$ T cells (% of CD45$^+$ cells)
*
Isotype + -
Anti-IL-10R - +
H
Relative IFN-γ mRNA expression
*
Isotype + -
Anti-IL-10R - +
T cells

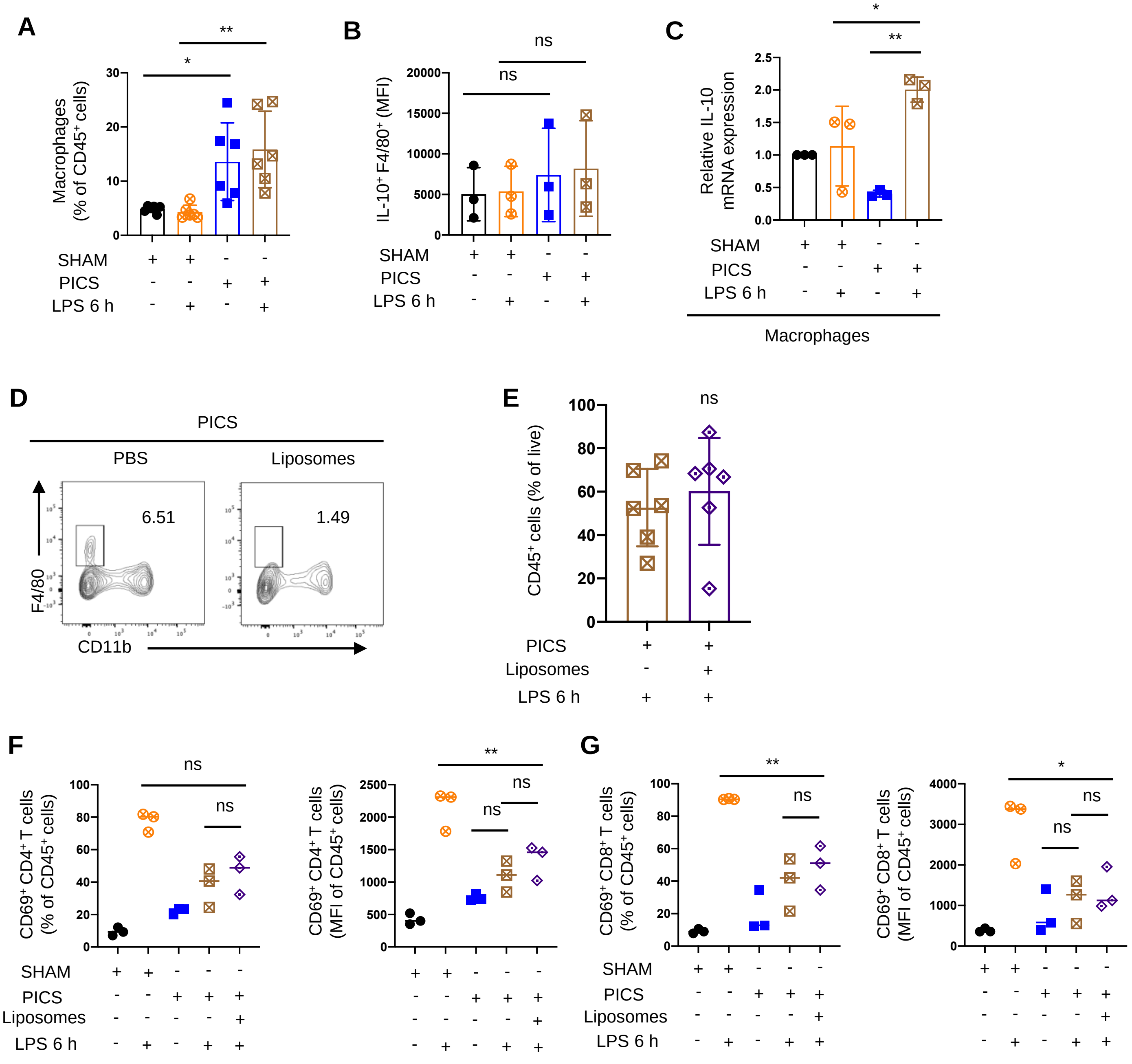

A
Macrophages (% of CD45+ cells)
30
20
10
0
*
**
SHAM + + - -
PICS - - + +
LPS 6 h - + - +
B
IL-10+ F4/80+ (MFI)
20000
15000
10000
5000
0
ns
ns
SHAM + + - -
PICS - - + +
LPS 6 h - + - +
C
Relative IL-10 mRNA expression
2.5
2.0
1.5
1.0
0.5
0.0
*
**
SHAM + + - -
PICS - - + +
LPS 6 h - + - +
Macrophages
D
PICS
PBS
Liposomes
6.51
1.49
F4/80
CD11b
E
CD45+ cells (% of live)
100
80
60
40
20
0
ns
PICS + +
Liposomes - +
LPS 6 h + +
F
CD69+ CD4+ T cells (% of CD45+ cells)
100
80
60
40
20
0
ns
ns
CD69+ CD4+ T cells (MFI of CD45+ cells)
2500
2000
1500
1000
500
0
**
ns
ns
SHAM + + - - -
PICS - - + + +
Liposomes - - - - +
LPS 6 h - + - + +
G
CD69+ CD8+ T cells (% of CD45+ cells)
100
80
60
40
20
0
**
ns
CD69+ CD8+ T cells (MFI of CD45+ cells)
4000
3000
2000
1000
0
*
ns
ns
SHAM + + - - -
PICS - - + + +
Liposomes - - - - +
LPS 6 h - + - + +

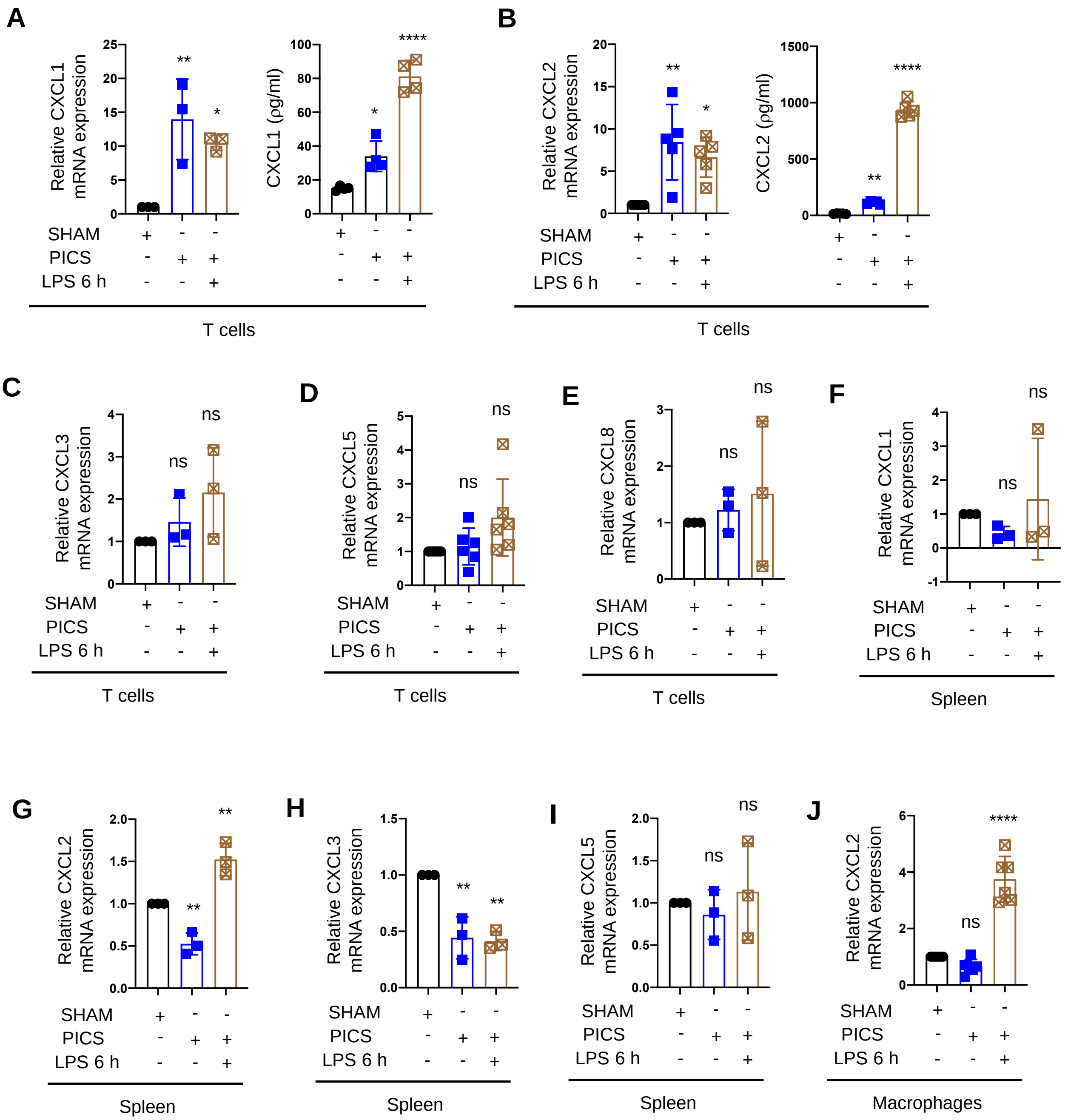

A
Relative CXCL1 mRNA expression
CXCL1 (ρg/ml)
SHAM
PICS
LPS 6 h
T cells
B
Relative CXCL2 mRNA expression
CXCL2 (ρg/ml)
T cells
C
Relative CXCL3 mRNA expression
T cells
D
Relative CXCL5 mRNA expression
T cells
E
Relative CXCL8 mRNA expression
T cells
F
Relative CXCL1 mRNA expression
Spleen
G
Relative CXCL2 mRNA expression
Spleen
H
Relative CXCL3 mRNA expression
Spleen
I
Relative CXCL5 mRNA expression
Spleen
J
Relative CXCL2 mRNA expression
Macrophages

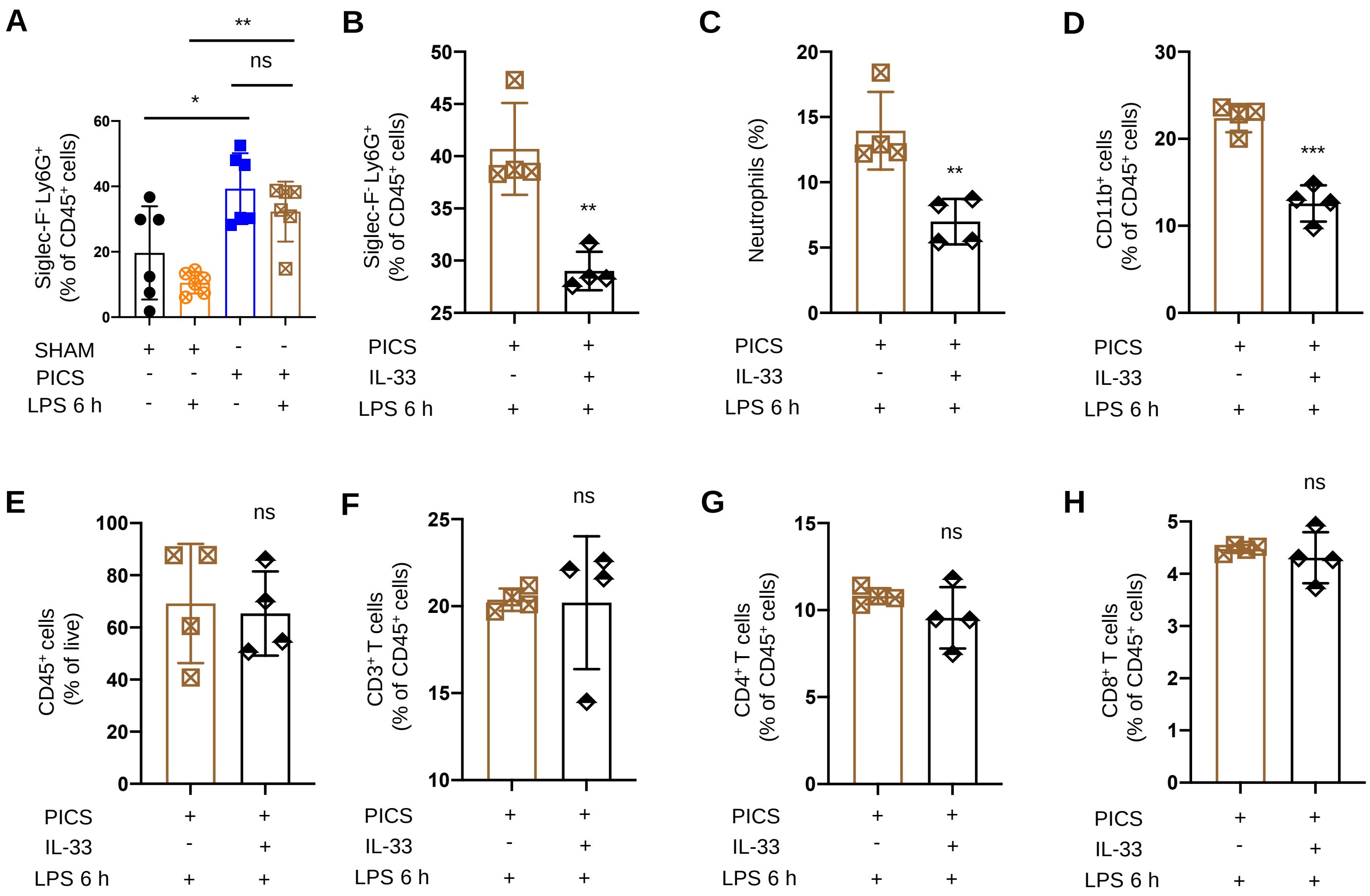

A
Siglec-F- Ly6G+ (% of CD45+ cells)
SHAM + + - -
PICS - - + +
LPS 6 h - + - +
*
**
ns
B
Siglec-F- Ly6G+ (% of CD45+ cells)
**
C
Neutrophils (%)
**
D
CD11b+ cells (% of CD45+ cells)
***
E
CD45+ cells (% of live)
ns
F
CD3+ T cells (% of CD45+ cells)
ns
G
CD4+ T cells (% of CD45+ cells)
ns
H
CD8+ T cells (% of CD45+ cells)
ns
PICS + +
IL-33 - +
LPS 6 h + +
